# Discovery of Inhibitory Active Ingredients for α-Amylase and α-Glucosidase from Raspberry (*Rubus idaeus* L.) Stems and Leaves Guided by Affinity Ultrafiltration and UPLC-QTOF-MS/MS

**DOI:** 10.3390/foods15071134

**Published:** 2026-03-25

**Authors:** Wei Zhao, Peng Yang, Mingyun Chen, Dongyu Gu, Dajun He

**Affiliations:** 1 Analysis and Testing Center, College of Life Sciences, Shihezi University, Shihezi 832000, China; vvzhao2023@163.com (W.Z.); yangpeng@stu.shzu.edu.cn (P.Y.); 13050137092@163.com (M.C.); 2 College of Marine Science and Environment, Dalian Ocean University, Dalian 116023, China; gudongyu@dlou.edu.cn

**Keywords:** *Rubus idaeus* L., α-glucosidase, α-amylase, inhibitors, UF-LC/MS, molecular docking

## Abstract

Raspberry (*Rubus idaeus* L.) fruits have been widely used due to their abundance of diverse polyphenolic compounds, whereas research on the chemical composition and bioactivity of their stems and leaves remains limited. In this study, the ethyl acetate extract of raspberry stems and leaves was evaluated for inhibitory activity against α-glucosidase and α-amylase. Guided by affinity ultrafiltration–mass spectrometry, 16 potential active components were further isolated and characterized. Among these, 13 compounds exhibited binding affinity for α-amylase, while 5 compounds showed binding affinity for α-glucosidase. Quercetin-3-*O*-β-D-glucoside-7-*O*-β-D-gentiobioside was isolated from raspberry stems and leaves for the first time. Procyanidin C_3_ and quercetin exhibited significant inhibitory effects on the two enzymes. Molecular docking studies hinted at the interactions between these compounds and the key active sites of the two enzymes. These findings suggest that phenolic compounds in raspberry stems and leaves may possess potential as α-glucosidase and α-amylase inhibitors, providing a scientific basis for further research on their application as functional components for blood glucose control.

## 1. Introduction

Diabetes mellitus (DM) is a widespread metabolic disease marked by high blood sugar, leading to significant disability and mortality [1,2]. Type II diabetes, the most common form, is linked to obesity and involves insulin resistance and reduced insulin secretion [3,4]. Natural enzyme inhibitors, such as phenolics and flavonoids, from various sources are used as alternative treatments for type 2 diabetes by targeting α-glucosidase and α-amylase [5,6]. Common drugs like acarbose, voglibose, and miglitol also serve this purpose but have notable side effects and do not fully prevent complications [4,7,8]. Therefore, it is important to search for natural active ingredients with hypoglycemic effects. Raspberry (*Rubus idaeus* L.) is a perennial shrub, which belongs to the genus *Rubus* and is widely distributed in the northeastern and northwestern areas of China [9,10].

Wu et al. analyzed the phenolic profiles of fruit, leaves, and seed extracts in raspberry, and 19 phenolic compounds were identified by HPLC-ESI-qTOF-MS/MS. The leaves extract of raspberry displayed the highest total phenolic content and total flavonoid content. The various parts of the raspberry had strong antioxidant activities and inhibitory effects on digestive enzymes [6]. Li et al. found the leaves extract of raspberry showed significant PTP1B inhibitory activity, and they tentatively identified 16 flavonoids in raspberry leaves by HPLC-qTOF-MS/MS [11]. Garjonyte et al. tentatively identified 10 phenolic compounds from *R. idaeus* stem by HPLC-DAD-TOF MS [12]. The extract of raspberry stem possesses a high polyphenol content and exhibits significant antioxidant activity. Subbiah et al. evaluated the phenolic content and antioxidant potential of raspberry fruit, and they tentatively identified 30 phenolic compounds in raspberry fruits by LC-ESI-QTOF-MS/MS [13]. These included procyanidin B_1_ and quercetin derivatives. These studies indicated that raspberries contain a diversity of phenolic compounds, exhibiting strong antioxidant and hypoglycemic activities. Previous studies have largely focused on identifying phenolic compounds in raspberry using liquid chromatography-mass spectrometry (LC-MS). There is a lack of research on the systematic isolation and identification of compounds from raspberries. Due to the limitations of mass spectrometry in qualitative analysis, it can only provide information on parent and fragment ions. Without comparison with mass spectral data of reference standard, structural identification is often unreliable.

Traditional research on bioactive natural products often employs a cyclic model of “extraction-isolation-bioassay,” where monomeric compounds are first isolated using chromatographic techniques and then subjected to bioactivity screening one by one. This strategy inherently presents challenges such as blind isolation procedures, heavy workload, long experimental cycles, and the potential loss of minor active components. To overcome these limitations, this study developed an integrated research strategy combining “affinity ultrafiltration-liquid chromatography/mass spectrometry (UF-LC/MS) targeted screening, bioactivity-guided isolation, and molecular docking validation.” The innovation of this approach is primarily reflected in the following two aspects. Firstly, UF-LC/MS technology was applied to raspberry for enzyme-guided fractionation, enabling rapid screening of α-amylase and α-glucosidase ligands from the extracts. This method, based on the specific binding between enzymes and ligands, enables the direct capture and identification of potential active compounds within a complex matrix, thereby avoiding the blindness of the traditional approach and significantly enhancing the efficiency and targeting of bioactive compound discovery. Secondly, molecular docking analysis revealed the structure–activity relationship between the active compounds and the target enzymes at the molecular level. The application of this integrated strategy offers a valuable methodological reference for the efficient discovery of active components from natural products.

Therefore, in order to rapidly and accurately discover α-glucosidase and α-amylase inhibitory active components in raspberry stems and leaves, we employed affinity ultrafiltration–LC-MS/MS as a guiding method to quickly screen for components with affinity for both enzymes. Subsequently, various chromatographic techniques were combined to isolate these potential active components. Through activity screening of isolated compounds and molecular docking studies, we aimed to identify the inhibitory active components for both enzymes in raspberry stems and leaves. This establishes a novel strategy for the efficient discovery of natural hypoglycemic lead compounds and provides a valuable reference for the subsequent development of anti-diabetic drug candidates.

## 2. Materials and Methods

### 2.1. Chemicals and Materials

α-Amylase (≥5 U/mg) and α-glucosidase (≥10 U/mg) were purchased from Sigma-Aldrich (St. Louis, MO, USA). Acarbose was provided by Shanghai Aladdin Biochemical Technology Co., Ltd. (Aladdin, Shanghai, China, purity > 98%). Maltose (HPLC) and xylitol (>99%) were purchased from Adamas-beta (Shanghai Titan Scientific Co., Ltd., Shanghai, China). Soluble starch (Acros Organics, Waltham, MA, USA) was used in the experiments. All chemical reference standards (anhydrous glucose, catechin, chlorogenic acid, procyanidin B_2_, quercetin-3-*O*-β-D-glucosyl-7-*O*-β-D-gentiobioside, caffeic acid, epicatechin, ellagic acid, rutin, hyperoside, quercetin-3-*O*-glucuronide, isoquercitrin, kaempferol-3-*O*-rutinoside, avicularin, astragalin, kaempferol-3-*O*-arabinoside, quercetin, and kaempferol) were obtained from Shanghai Tauto Biotech Co., Ltd. (Shanghai, China).

### 2.2. Extraction

10.85 kg of raspberry stems and leaves were air-dried. The material was soaked in methanol at a ratio of 1:10 at room temperature for 24 h. The soaking solution was collected, and the extraction was repeated until no significant color change was observed. The combined extracts were concentrated under reduced pressure at 40 °C using a rotary evaporator (Heidolph, Schwabach, Germany) to obtain the crude extract (1.85 kg, with a yield of 17.05%). After preparing an aqueous suspension of the extract, it was sequentially extracted with equal volumes of petroleum ether, dichloromethane, ethyl acetate, and *n*-butanol until the organic layer became clear and nearly colorless. After collecting each organic layer and the aqueous layer separately, they were concentrated under reduced pressure to obtain the petroleum ether fraction (904.0 g, with a yield of 48.86%), dichloromethane fraction (45.2 g, with a yield of 2.45%), ethyl acetate fraction (96.9 g, with a yield of 5.24%), *n*-butanol fraction (84.2 g, with a yield of 4.55%), and aqueous fraction (509.0 g, with a yield of 27.52%), the sum of the extract masses from all fractions accounted for 88.62% of the crude extract.

### 2.3. Analysis Activity of α-Amylase

Referring to the method with modifications [14], an ammonium acetate buffer (pH 6.86, 10 mM) was used as the reaction system, and the sample was dissolved in a 10% DMSO aqueous solution. α-Amylase was prepared as a 3.75 U/mL enzyme solution using the buffer. The substrate was a 1% soluble starch solution which was dissolved in the buffer. The experiment included four groups: blank group (BG), sample group (experimental group—EG), blank control group (BCG), and sample control group (SCG). The procedure for the sample group was as follows: 100 μL of α-amylase solution (3.75 U/mL) was mixed with 100 μL of sample solution (six concentration gradients, three replicates each). The mixture was incubated at 37 °C for 15 min, followed by adding 100 μL of starch solution and reacting at room temperature for 10 min. Subsequently, 200 μL of DNS reagent was added, and the mixture was heated in a boiling water bath for 5 min. After cooling to room temperature, 1.5 mL of deionized water was added for dilution. The solution was vortexed, and 200 μL of the reaction mixture was transferred to a 96-well plate to measure the absorbance at 540 nm by the microplate reader (Multiskan FC, Thermo Fisher Scientific Instrument Co., Ltd., Shanghai, China). Both the BG and the BCG used a 10% DMSO buffer solution instead of the sample solution. The BCG and the EG used PBS instead of the enzyme solution. Acarbose was used as the positive control. The inhibition rate was calculated according to the following Formula (1):

(1) Inhibition rate (%) = [1 − (A_1_ − A_2_)/(A_3_ − A_4_)] × 100

where A_1_, A_2_, A_3_, and A_4_ represent the absorbance values at 540 nm for the EG, SCG, BG, and BGC, respectively. The calibration curve was plotted using a glucose standard (0.1–2.0 mg/mL), with a linear regression equation of *y* = 1.031 *x* − 0.0216 and *R*^2^ = 0.9955, and the IC_50_ value was calculated using IBM SPSS Statistics 26.0.

### 2.4. Analysis Activity of α-Glucosidase

Referring to method [15] with slight modifications, an ammonium acetate buffer (pH 6.86, 10 mM) was used as the reaction system. α-Glucosidase was prepared as a 1.0 U/mL enzyme solution using the buffer. The experiment was divided into four groups: blank group (BG), blank control group (BCG), sample control group (SCG), and sample group (experimental group—EG). The procedure for the experimental group was as follows: 40 μL of α-glucosidase solution (1.0 U/mL) was mixed with 40 μL of sample solution (six concentrations, three replicates) and incubated at 37 °C for 30 min. Then, 20 μL of maltose substrate (0.5 mg/mL) was added, and the mixture was incubated at 37 °C for 20 min. The reaction was stopped by adding 895 μL of methanol, with 5 μL of xylitol added as an internal standard. Finally, the mixture was centrifuged at 10,000 rpm and 20 °C for 10 min, filtered through a 0.22 μm membrane, and analyzed using LC-ESI-MS/MS. Both the BG and the BCG used 10% DMSO ammonium acetate solution instead of the sample solution. The BCG and the EG used ammonium acetate buffer instead of the enzyme solution. Acarbose served as the positive control. The inhibition rate was calculated using the following Formula (2):

(2) Inhibition rate (%) = [1 − (RC_1_ − RC_2_)/(RC_3_ − RC_4_)] × 100

where RC_1_, RC_2_, RC_3_, and RC_4_ represent the maltose concentrations generated by the SCG, EG, BCG and BG, respectively.

An ACQUITY UPLC system coupled with a triple quadrupole tandem mass spectrometer (Xevo TQS, Waters Corp., Milford, MA, USA) was used to determine the glucose content. Electrospray ionization (ESI) was applied with an ion source temperature of 150 °C and a capillary voltage of 2.0 kV. The desolvation gas temperature was set at 450 °C with a flow rate of 800 L/h, while the cone gas flow rate was 150 L/h. The nebulizer gas pressure was maintained at 7.0 bar, and detection was performed in multiple reaction monitoring (MRM) mode. An ACQUITY UPLC BEH Amide column (1.7 μm, 2.1 × 50 mm) was used. The mobile phase consisted of water (A) and acetonitrile (B). The elution gradient was as follows: 0 min, 30% A; 0–1.0 min, 30–45% A; 1.0–2.0 min, 45–50% A; 2.0–3.0 min, 50% A; 3.0–3.5 min, 50–30% A; 3.5–5.0 min, 30% A. The mass spectrometry parameters for glucose, xylitol, and maltose are provided in Supplementary Material Table S1. Intra-day precision, inter-day precision, and accuracy were tested by measuring maltose at different concentrations. Precision is expressed as relative standard deviation (RSD) is within 15%, and accuracy is expressed as relative error (RE) does not exceed 15% of the actual sample value (Tables S2 and S3). The stability and reliability of maltose measurement were also validated. The linearity of maltose quantification was established over 0.05–10 μg/mL (*y* = 72.1289 *x* + 90.5583, *R*^2^ = 0.9990).

### 2.5. Screening and Identification of Potential Inhibitors

#### 2.5.1. UPLC-PDA-qTOF-MS/MS Analysis of *R. idaeus* Extracts

An ACQUITY UPLC system coupled with a quadrupole time-of-flight mass spectrometer (SYNAPT XS, Waters Corp., Milford, MA, USA) was used. The optimized UPLC conditions were as follows: the ACQUITY PREMIER^®^ HSS T3 column (1.8 μm, 2.1 mm × 10 cm, Waters, USA) was used. The mobile phase A was 0.1% formic acid in water, and mobile phase B was acetonitrile. The injection volume was 1.00 μL, the column temperature was set at 40 °C, and the flow rate was 0.3 mL/min. The mobile phase gradient program was as follows: 0–1.0 min, 98% A; 1.0–3.0 min, 95% A; 3.0–5.0 min, 90% A; 5.0–14.0 min, 86% A; 14.0–16.0 min, 80%A; 16.0–18.0 min, 70% A; 18.0–23.0 min, 50% A; 23.0–25.2 min, 0% A; 25.2–28.0 min, 98% A. MS analysis was performed in negative ion (ES^−^) MS^e^ scanning mode. The optimized mass spectrometry parameters were as follows: ion source temperature 120 °C; capillary voltage 1 kV; desolvation temperature 500 °C; desolvation gas flow 1000 L/h; cone gas flow 50 L/h; nebulizer gas pressure 6.5 bar. Data acquisition and processing were performed using Waters Mass Lynx v4.1 software.

#### 2.5.2. Rapid Screening of Potential α-Amylase Inhibitors with UF-LC/MS

Referring to method [16,17] with modifications, α-amylase with a concentration of 3.75 U/mL (dissolved in ammonium acetate buffer, 10 mM, pH 6.86) was used. The ethyl acetate fraction of the raspberry had a concentration of 10 mg/mL (dissolved in 10% DMSO). The enzyme solution and sample solution were mixed at a ratio of 800 μL: 200 μL and incubated at 37 °C for 30 min. The mixture was then transferred to a centrifugal filter unit (4 mL, BIOFIL, Guangzhou, China) and centrifuged (20 °C, 4800× *g*, 15 min) to remove the filtrate. After washing three times with buffer followed by centrifugation, the residue was dissociated with 1 mL of 90% aqueous methanol for 10 min (repeated three times), then centrifuged again, filtered through a 0.22 μm membrane, and analyzed by LC/MS. Three types of controls were used: (i) active enzyme (experimental group), (ii) inactivated enzyme (boiled for 30 min) to account for non-specific binding to denatured proteins, and (iii) ammonium acetate buffer replacing the enzyme solution to account for non-specific adsorption to the ultrafiltration membrane. All experiments were performed in triplicate.

An inactivated α-AMY was taken as control. Binding ratio (BR) of the ligands and α-AMY was calculated according to the Formula (3):

(3) Binding ratio (BR, %) = (A_1_ − A_2_)/A_0_ × 100

where A_0_ is the peak area of each compound of the samples before ultrafiltration; A_1_ and A_2_ are the peak area of the bound compounds by activated enzymes and inactivated enzymes after ultrafiltration, respectively. This calculation corrects for non-specific binding and normalizes to initial compound abundance. Compounds with a BR greater than 1% were considered as potential α-amylase ligands for further analysis. The relative standard deviation (RSD) of BR values for triplicate experiments was less than 5%, indicating acceptable method repeatability.

#### 2.5.3. Rapid Screening of Potential α-Glucosidase Inhibitors with UF-LC/MS

Referring to method [18,19] with modifications, α-glucosidase at a concentration of 5 U/mL (dissolved in ammonium acetate buffer, 10 mM, pH 6.86) was used. The sample concentration was 2 mg/mL (dissolved in 10% DMSO). The enzyme solution and sample solution were mixed at a ratio of 600 μL: 500 μL and incubated at 37 °C for 20 min. The mixture was transferred to an ultrafiltration unit (4 mL, BIOFIL), and centrifuged (20 °C, 4800× *g*, 15 min) to remove the filtrate. After three washes with buffer followed by centrifugation, the residue was dissociated with 1 mL of 90% aqueous methanol for 10 min and centrifuged. This step was repeated three times. The collected filtrates were combined, dried under nitrogen, redissolved in 100 μL of methanol, centrifuged, filtered through a 0.22 μm membrane, and analyzed by LC/MS. The same control groups (active enzyme, inactivated enzyme, and buffer) and triplicate measurements were applied as described in Section 2.5.2. Compounds with a BR greater than 1% were considered as potential α-glucosidase ligands for further analysis. The relative standard deviation (RSD) of BR values for triplicate experiments was less than 5%, indicating acceptable method repeatability.

### 2.6. Isolation and Structural Identification of Active Compounds

96.9 g of ethyl acetate fraction from raspberry stems and leaves was separated by silica gel (200–300 mesh) column (150 mm ×10 cm), followed by gradient elution with dichloromethane: methanol (100%:0~0:100%). Each 150 mL portion was collected as a fraction. After concentration under reduced pressure and identification by TLC, 11 fractions were obtained. Different fractions were subsequently subjected to separation and purification using a Sephadex LH-20 gel column (20 mm × 200 cm, GE Healthcare, Uppsala, Sweden). Elution was carried out with methanol (0.1% FA), and each 10 mL portion was collected as a fraction. Isolated compounds were identified by TLC, UPLC-QTOF-MS/MS and nuclear magnetic resonance (NMR) spectrometer (Bruker, 400 MHz, Karlsruhe, Germany).

### 2.7. Activity Screening of Isolated Compounds

Referring to the method described in Section 2.3 and combining the results of affinity ultrafiltration, the in vitro activity of the isolated compounds was determined, and their IC_50_ values were calculated.

### 2.8. Molecular Docking Study

**Figure 1:**
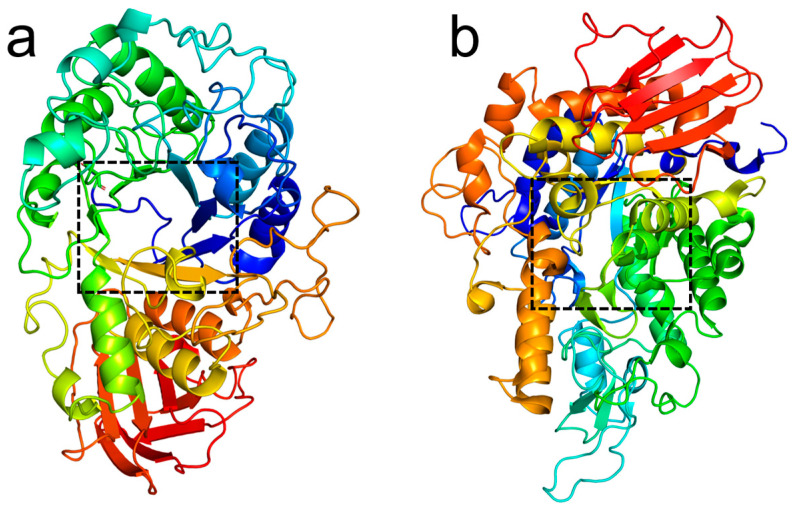
Schematic representation of enzymes. (**a**) α-amylase (1OSE), and (**b**) α-glucosidase (3A4A).

The selected compounds exhibited optimal inhibitory activity against either α-amylase or α-glucosidase, or demonstrated dual inhibitory efficacy. Molecular docking analysis was performed using these compounds. The three-dimensional structure of α-amylase (PDB ID: 1OSE) and α-glucosidase (PDB ID: 3A4A) were retrieved from the Protein Data Bank (PDB, https://www.rcsb.org/) as shown in Figure 1. Ligand structures were obtained from the PubChem database (https://pubchem.ncbi.nlm.nih.gov/) [15,20]. Molecular docking and minimization were conducted using AutoDock 1.5.7. Visualization and analysis of docking poses were performed with PyMOL 2.6. The docking grid boxes were defined to cover the entire active site of each enzyme. An exhaustiveness of 32 was used, and 10 binding modes were generated for each ligand. The conformation with the lowest binding energy was selected for further analysis. The docking protocol was validated by redocking the co-crystallized ligands into their respective binding sites, RMSD < 2.0 Å.

### 2.9. Data Processing and Analysis

The IC_50_ values were calculated using SPSS. Figures were generated with Origin 2024.

## 3. Results

### 3.1. Inhibitory Activity of Different Extraction Fractions of Raspberry on α-Amylase and α-Glucosidase

The results of in vitro inhibition assays (Table 1) showed that the ethyl acetate fraction exhibited the lowest IC_50_ values against both α-amylase and α-glucosidase among the four extract fractions of raspberry stem and leaf, indicating it possessed the strongest inhibitory effect. The ethyl acetate fraction (IC_50_ = 34.49 ± 0.84 μg/mL) showed even better inhibition against α-glucosidase than the positive control acarbose (IC_50_ = 93.30 ± 2.12 μg/mL). This was followed by the *n*-Butanol fraction, the aqueous fraction, and the dichloromethane fraction. Meanwhile, UF-LC/MS screening revealed that the ethyl acetate fraction was most enriched in potential enzyme ligands. Then, the ethyl acetate fraction was chosen for further isolation and identification of inhibitors targeting α-amylase and α-glucosidase.

### 3.2. Analysis of Affinity Ultrafiltration

**Figure 2:**
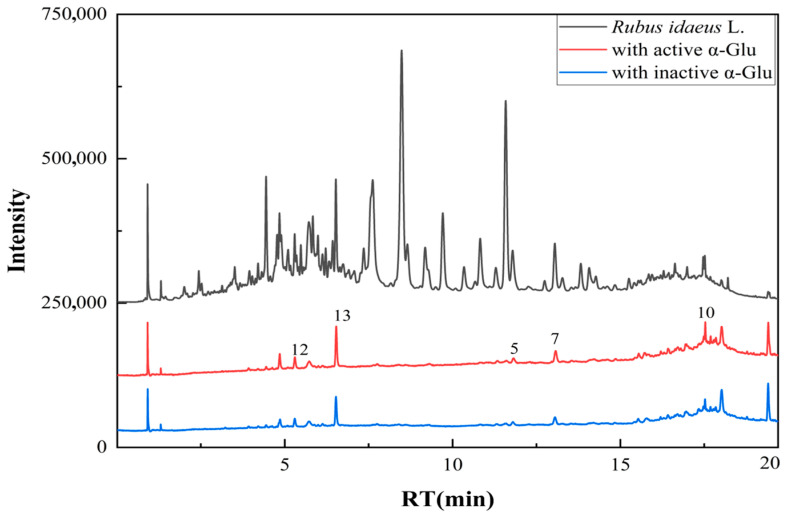
PDA chromatograms of the affinity ultrafiltration results for raspberry stem and leaf extract with α-glucosidase.

**Figure 3:**
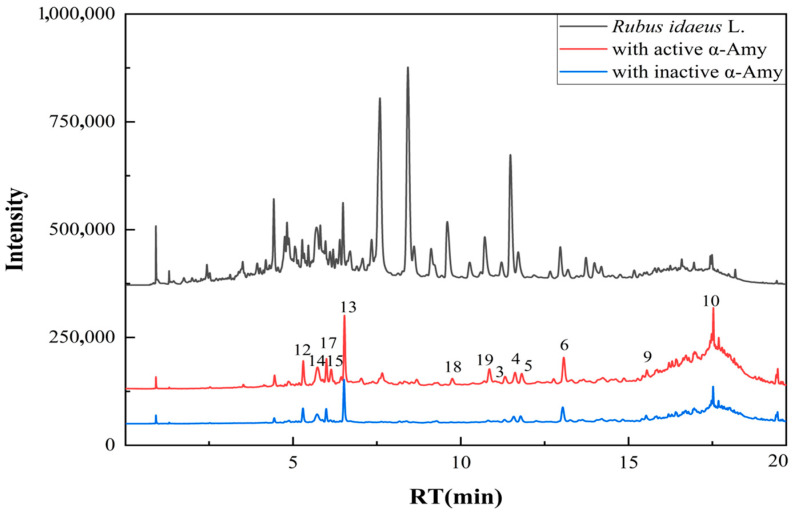
PDA chromatograms of the affinity ultrafiltration results for raspberry stem and leaf extract with α-amylase.

**Figure 4:**
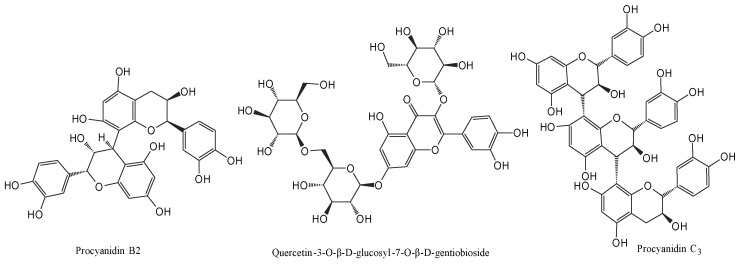
Structure of the isolated compounds from raspberry leaves and stems.

This study employed affinity ultrafiltration coupled with UPLC-MS/MS technology to screen potential α-amylase inhibitors (α-AIs) and α-glucosidase inhibitors (α-GIs) in the ethyl acetate fraction of raspberry stem and leaf extract. The sample was incubated with α-amylase and α-glucosidase separately, followed by retention through an ultrafiltration membrane. Compounds bound to the enzymes were ultimately analyzed using the UPLC-MS/MS method. 13 compounds showed specific binding to α-amylase, and 5 compounds exhibited specific binding to α-glucosidase (Figure 2 and Figure 3). Based on the UPLC-MS/MS results and compared with the MS information of the standard compound, a total of 19 compounds were tentatively identified (Table 2). The affinity ultrafiltration results indicated that quercetin (the binding rate (BR) = 14.88%), procyanidin C_3_ (BR = 8.85%), kaempferol-3-*O*-arabinoside (BR = 5.42%), catechin (BR = 4.96%), ellagic acid (BR = 3.84%), and hyperoside (BR = 3.64%) exhibited relatively high binding rates to α-amylase. In comparison, caffeic acid, epicatechin, isoquercitrin, and quercetin-3-*O*-glucuronide showed lower binding rates to α-amylase. In the binding to α-glucosidase, the binding rates were as follows: quercetin (BR = 8.88%) > epicatechin (BR = 2.42%) > isoquercitrin (BR = 1.94%) > catechin (BR = 1.73%) > avicularin (BR = 1.60%). Five main components: catechin, epicatechin, isoquercitrin, avicularin, and quercetin exhibited binding affinity to both α-amylase and α-glucosidase. Quercetin ranked highest in binding to both enzymes, it may be a key compound responsible for the hypoglycemic activity of the raspberry stem and leaf extract. Partial complex compound structures are shown in Figure 4.

### 3.3. Isolation and Identification of Compounds

In this study, silica gel column chromatography combined with gel column separation were employed for the isolation and identification of compounds. A total of 16 compounds were isolated. Among them, using silica gel column separation combined with gel column purification, compound 1 (25.60 mg) and compound 2 (21.14 mg) were isolated from Fr.4; compound 3 (33.55 mg) and compound 8 (36.65 mg) were isolated from Fr.5; compound 4 (56.60 mg), compound 5 (43.35 mg), and compound 9 (21.44 mg) were isolated from Fr.6; compound 6 (27.76 mg) and compound 7 (24.5 mg) were isolated from Fr.7, compound 10 (14.50 mg) and compound 13 (1.53 mg) were from Fr.6, compound 11 (28.60 mg) and compound 12 (3.30 mg) were obtained isolated from Fr.8, compound 14 (11.03 mg) was isolated from Fr.7, compound 15 (7.86 mg) and compound 16 (31.20 mg) was obtained from Fr.3. The structure of isolated compounds were identified by NMR spectroscopy and UPLC-QTOF-MS/MS analysis.

According to the MS information and NMR spectrum and compared with literature data, the compounds (**1**–**11**) were identified as **kaempferol** (**1**) [21,22], **ellagic acid** (**2**) [23,24], **rutin**(**3**) [25], **hyperoside**(**4**) [26], **isoquercitrin** (**5**) [27,28], **epicatechin** (**6**) [29], **catechin** (**7**) [30], **quercetin**(**8**) [31], **caffeic acid** (**9**) [32], **astragalin** (**10**) [33], **kaempferol-3-*****O*****-rutinoside** (**11**) [34]. The NMR and mass spectrometry data are available in the Supplementary Materials. Compounds **12**, **13**, **15** were identified as **kaempferol-3-*****O*****-arabinoside** (**12**), **quercetin-3-*****O*****-glucuronide** (**13**), **chlorogenic acid** (**15**) by comparison with high-resolution mass spectrometry (HR-MS) data of the reference standard [35,36,37].

Compound **14**: C_33_H_40_O_22_, yellow powder, ESI-MS [M-H]^−^ m/z: 787.1406; ^1^H-NMR (MeOD-d_4_) *δ*: 12.73 (1H, S, 5-OH), 9.42 (2H, S, 4′-OH, 3′-OH), 7.71 (1H, d, *J* = 2.2 Hz, H-2′), 7.65 (1H, dd, *J* = 2.2, 8.4 Hz, H-6′), 6.94 (1H, d, *J* = 8.5 Hz, H-5′), 6.85 (1H, d, *J* = 2.1 Hz, H-8), 6.57 (1H, d, *J* = 2.1 Hz, H-6), 5.55 (1H, d, *J* = 7.0 Hz, 3-glc-H-1), 5.18 (1H, d, *J* = 6.8 Hz, 7-glc-H-1), 4.24 (1H, d, *J* = 7.7 Hz, 7-glc-glc-H-1), 3.0~4.1 (m, sugar-H); ^13^C NMR (400 MHz, MeOD): 177.66 (C-4), 162.84 (C-7), 160.98 (C-5), 156.84 (C-2), 155.98 (C-9), 148.70 (C-4′), 144.82 (C-3′), 133.61 (C-3), 121.67 (C-6′), 121.08 (C-1′), 116.59 (C-2′), 115.32 (C-5′), 105.70 (C-10), 103.54 (7-glc-glc-C1′′′′), 100.80 (3-glc-C1′′), 99.75 (7-glc-glc-C1′′′), 94.46 (C-8), 77.63 (3-glc-C5′′), 77.03 (7-glc-glc-C5′′′′), 76.77 (3-glc-C3′′), 76.54 (7-glc-glc-C3′′′′), 76.28 (7-glc-glc-C3′′′), 75.37 (7-glc-glc-C5′′′), 73.49 (7-glc-glc-C2′′′′), 74.13 (3-glc-C2′′), 73.13 (7-glc-glc-C2′′′), 69.95 (7-glc-glc-C4′′′′), 69.28 (3-glc-C4′′), 69.47 (7-glc-glc-C4′′′), 61.10 (7-glc-glc-C6′′′′), 60.98 (3-glc-C6′′). Compared with the HRMS and NMR data with previous studies, compound **14** was identified as **quercetin-3-*****O*****-β-D-glucopyranosyl-7-*****O*****-β-D-gentiobioside** [38].

Compound **16**: C_45_H_38_O_18_, brown crystalline solid. ESI-MS [M-H]^−^ m/z: 865.1999; ^1^H-NMR (MeOD-d_4_) *δ*: 2.35 (s, 1H, H-4′′ β), 2.41 (d, *J* = 16.5 Hz, 1H, H-4′′ α), 3.65 (d, *J* = 5.7 Hz, 1H, H-3), 3.93 (m, 1H, H-3′′), 4.04 (d, *J* = 9.1 Hz, 1H, H-2′), 4.09 (d, *J* = 7.5 Hz, 1H, H-3′), 4.16–4.24 (m, 1H, H-4′), 4.32 (d, *J* = 7.0 Hz, 1H, H-4), 4.37 (d, *J* = 7.2 Hz, 1H, H-2), 5.45 (d, *J* = 2.4 Hz, 1H, H-6′), 5.52 (d, *J* = 10.1 Hz, 2H, H-6, H-8), 5.74 (s, 1H, H-6′′), 6.82 (d, *J* = 7.6 Hz, 1H, H-14), 6.68 (t, *J* = 2.1 Hz, 2H, H-13′′, H-13), 6.62–6.48 (m, 3H, H-13′, H-14′, H-14′′), 7.54 (s, 1H, H-10′′), 8.01 (s, 1H, H-10), 8.10 (d, *J* = 13.6 Hz, 2H, H-10′); ^13^C NMR (100 MHz, DMSO-d_6_): 157.48 (C-7), 156.81 (C-5), 156.33 (C-8), 155.62 (C-8′), 154.43 (C-5′), 154.10 (C-7′), 153.74 (C-5′′), 153.46 (C-7′′), 153.20 (C-8′′), 145.01 (C-12′′), 144.93 (C-11′), 144.85 (C-12′), 144.70 (C-11′′), 144.59 (C-12), 144.39 (C-11), 131.47 (C-9), 131.07 (C-9′′), 131.00 (C-9′), 130.90 (C-14′), 119.90 (C-14′′), 119.63 (C-14), 118.11 (C-10′), 115.89 (C-13), 115.38 (C-13′′), 114.98 (C-13′), 114.90 (C-10), 114.84 (C-10′′), 108.82 (C-8′), 108.78 (C-8′′), 106.44 (C-4′), 105.82 (C-4, C-4′′), 97.99 (C-6′′), 96.93 (C-6), 96.07 (C-6′), 94.15 (C-8), 82.73 (C-2′), 82.60 (C-2′′), 78.35 (C-2), 71.58 (C-3′), 71.28 (C-3), 65.20 (C-3′′), 38.03 (C-4′), 37.57 (C-4), 29.45 (C-4′′). Compared with the HRMS and NMR data with previous studies, compound **16** was identified as **procyanidin C_3_** [14].

### 3.4. Inhibitory effect of Isolated Compounds on α-Glucosidase and α-Amylase 

To further validate the activity, this study performed in vitro enzyme inhibition assays and determined the IC_50_ values of each isolated compound (Table 2). The results showed that procyanidin C_3_ exhibited the strongest inhibitory activity against α-amylase (IC_50_ = 78.67 ± 2.31 μg/mL), slightly surpassing acarbose (IC_50_ = 97.63 ± 8.00 μg/mL). This was followed by epicatechin (IC_50_ = 94.33 ± 5.51 μg/mL) and catechin (IC_50_ = 100.67 ± 5.51 μg/mL). Regarding α-glucosidase inhibition, acarbose had an IC_50_ = 93.30 ± 2.12 μg/mL, while procyanidin C_3_ (IC_50_ = 46.98 ± 4.43 μg/mL) remained significantly more potent than all other tested natural compounds. Among the other compounds, caffeic acid, astragalin, and quercetin-3-*O*-glucuronide also showed considerable inhibitory activity against α-glucosidase, with IC_50_ values ranging from 150 to 190 μg/mL. Quercetin exhibited notable dual inhibitory activity against both enzymes, with IC_50_ values below 260 μg/mL. However, no simple positive correlation was observed between inhibitory activity and the binding rate (BR) from affinity ultrafiltration. For example, kaempferol-3-*O*-arabinoside displayed a relatively high BR among all compounds, yet its IC_50_ exceeded 1000 μg/mL. This suggests that strong initial binding affinity does not necessarily lead to high functional inhibitory efficacy. The discrepancy may arise from binding at non-critical active sites or from binding modes that fail to effectively block the catalytic reaction.

### 3.5. Molecular Docking Analysis

Molecular docking is a key method for studying the binding sites of small-molecule inhibitors with receptor enzymes, which aids in further elucidating their inhibitory mechanisms. Docking results of the positive control acarbose showed binding energies of −9.09 kcal/mol toward α-amylase and −9.08 kcal/mol toward α-glucosidase, with 15 and 18 hydrogen-bond interactions, respectively. These results (Table 3) are consistent with those reported in the literature [39,40,41].

**Figure 5:**
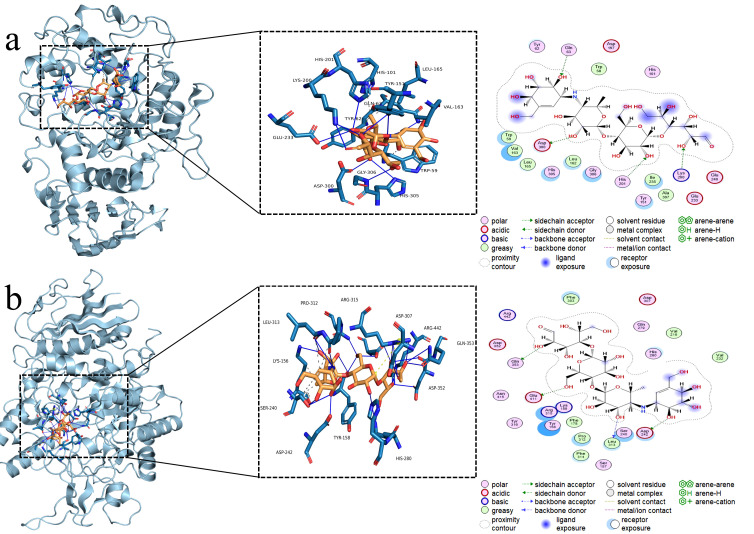
The docking conformations and the detailed interactions of α-amylase (**a**) and α-glucosidase (**b**) with acarbose. (Dashed blue line represents hydrogen-bond interaction. Blue green represents the functional amino acid residues, while yellow represents acarbose molecules. The light yellow dashed line represents disulfide bonds.)

In terms of structural classification, flavonols generally exhibited strong binding affinity. Among them, quercetin-3-*O*-β-D-glucoside-7-*O*-β-D-gentiobioside displayed binding energies of −9.55 kcal/mol and −10.36 kcal/mol toward α-amylase and α-glucosidase, forming 11 and 21 hydrogen bonds, respectively, representing the strongest binding flavonol. Rutin formed up to 22 hydrogen bonds with α-glucosidase (Figure 5), with a binding energy of −9.56 kcal/mol, indicating an extensive interaction network. Notably, flavonol aglycones showed relatively lower binding energies: quercetin exhibited −5.79 kcal/mol and −5.85 kcal/mol, and kaempferol −5.64 kcal/mol and −6.06 kcal/mol for the two enzymes, suggesting that glycosylation significantly enhances binding affinity to the enzyme active sites. Catechin and epicatechin showed moderate binding energies ranging from −5.7 to −6.2 kcal/mol, with 7–14 hydrogen bonds. Epicatechin showed a slightly higher binding affinity toward α-amylase (−6.15 kcal/mol) than catechin (−5.71 kcal/mol), consistent with the in vitro activity trend in which epicatechin exhibited stronger α-amylase inhibitory activity (IC_50_ 94.33 ± 5.51 μg/mL vs. 100.67 ± 5.51 μg/mL). Proanthocyanidins exhibited the strongest binding capacity. Procyanidin C_3_ achieved binding energies of −9.33 kcal/mol and −10.45 kcal/mol toward α-amylase and α-glucosidase, forming 24 and 14 hydrogen bonds, respectively, representing the lowest binding energy and most intensive hydrogen-bond network among all tested compounds. This result is highly consistent with its in vitro activities (IC_50_ 78.67 ± 2.31 μg/mL against α-amylase and 46.98 ± 4.43 μg/mL against α-glucosidase), confirming its great potential as a dual enzyme inhibitor. Procyanidin B_2_ also showed strong binding, with −7.41 kcal/mol and −8.68 kcal/mol for the two enzymes. Among ellagic acid and its derivatives, ellagic acid showed moderate binding, with −5.42 kcal/mol and −5.91 kcal/mol and 6–8 hydrogen bonds.

Analysis of key amino acid residues revealed that residues TRP-59, TYR-151, ASP-197, LYS-200, HIS-201, GLU-233, HIS-299, and ASP-300 in the active site of α-amylase were frequently involved in hydrogen bonding and served as common targets for most compounds. For α-glucosidase, major interaction residues included LYS-156, TYR-158, SER-240, ASP-242, HIS-280, ASP-307, PRO-312, ARG-315, and GLU-411. Procyanidin C_3_ formed a dense hydrogen-bond network with key α-amylase residues including TRP-59, GLN-63, TYR-151, LYS-200, GLU-233, and ASP-300, similar to the binding mode of acarbose, providing a molecular explanation for its strong inhibitory activity.

## 4. Conclusions

This study adopted an integrated strategy of “affinity ultrafiltration screening-targeted isolation-activity validation” to rapidly identify and isolate a series of α-amylase and α-glucosidase inhibitors from raspberry stems and leaves. Quercetin (BR for α-amylase: 14.88%, IC_50_: 258.67 ± 10.79 μg/mL; BR for α-glucosidase: 8.88%, IC_50_: 230.88 ± 7.83 μg/mL) and procyanidin C_3_ (BR for α-amylase: 8.85%, IC_50_: 78.67 ± 2.31 μg/mL; IC_50_ for α-glucosidase: 46.98 ± 4.43 μg/mL) showed particularly prominent activity. Their inhibitory effects were close to or superior to those of acarbose. Molecular docking offering plausible explanations for observed inhibitory activities, these compounds mainly achieve inhibition by forming a stable hydrogen-bond network with key residues in the active sites, such as TRP-59, TYR-151, ASP-197, LYS-200, HIS-201, GLU-233, HIS-299, ASP-300 in the active site of α-amylase, and major interaction residues of α-glucosidase included LYS-156, TYR-158, SER-240, ASP-242, HIS-280, ASP-307, and so on. Furthermore, the crude extract exhibited higher activity than the isolated compounds. This phenomenon suggests that there may be mixing effects, or that some highly active but low-abundance compounds have not been isolated. This work highlights the resource value of raspberry stems and leaves as functional ingredients for hypoglycemic purposes. It also verifies the effectiveness of the affinity ultrafiltration-guided screening strategy. The findings provide a basis for the development of natural dual inhibitors of α-amylase and α-glucosidase. Although this study discovered several compounds with significant inhibitory activities against α-amylase and α-glucosidase from raspberry stems and leaves, numerous avenues remain for further in-depth investigation in this study. Firstly, all activity evaluations were based on in vitro enzyme inhibition assays, lacking in vivo validation using cellular models and animal models. Therefore, the actual hypoglycemic effects, bioavailability, metabolic stability, and potential toxic side effects of these compounds remain unclear. Secondly, the molecular docking results only provide theoretical binding modes, which have not been validated through experimental techniques such as surface plasmon resonance (SPR), isothermal titration calorimetry (ITC), or site-directed mutagenesis. Thirdly, pharmacokinetic studies and safety evaluations of the active compounds were not conducted, which limits their further development and application. Future research should incorporate diabetic animal models, pharmacokinetic experiments, and toxicity evaluations to comprehensively assess the in vivo efficacy and safety of these compounds. Furthermore, the stability of these active compounds during food processing and their bioaccessibility under simulated gastrointestinal conditions should be evaluated to substantiate their potential as functional food ingredients. This would lay a foundation for their application in the development of functional foods or pharmaceuticals.

## Figures and Tables

**Table 1:** Inhibitory Activity of Different Extraction Fractions of Raspberry.

	Name	α-Amylase (μg/mL)	α-Glucosidase (μg/mL)
Positive Control	Acarbose	97.63 ± 8.00	93.30 ± 2.12
Raspberry Stem and Leaf Extract	Dichloromethane fraction	/	93.90 ± 1.96
	Ethyl acetate fraction	175.08 ± 4.90	34.49 ± 0.84
	*n*-Butanol fraction	286.66 ± 10.57	41.07 ± 3.21
	Aqueous fraction	/	60.71 ± 1.65

**Table 2:** Identification of active compounds from the ethyl acetate fraction of raspberry stems and leaves.

NO.	RT	M_W_	[M-H]^-^	MS/MS	Molecular Formula	Compounds	BR (%)		IC_50_ (μg/ mL)	
							α-Amy	α-Glu	α-AIA	α-GIA
Positive Control										
						Acarbose	/	/	97.63 ± 8.00	93.30 ± 2.12
Flavonols										
1	5.01	788	787.1406	787.1406, 301.0341, 135.0451	C_33_H_40_O_22_	Quercetin-3-*O*-β-D-glucosyl-7-*O*-β-D-gentiobioside	/	/	>1000	>500
2	11.10	610	609.1487	609.1487, 301.0348	C_27_H_30_O_16_	Rutin	/	/	562.00 ± 20.42	139.14 ± 10.52
3	11.40	464	463.0875	463.0883, 300.0292, 271.0223, 255.0302	C_21_H_20_O_12_	Hyperoside	3.64%	/	581.33 ± 32.32	316.99 ± 16.16
4	11.60	478	477.0671	451.3287, 301.0348, 178.997	C_21_H_18_O_13_	Quercetin-3-glucuronide	1.04%	/	374.33 ± 132.58	188.48 ± 7.89
5	11.80	464	463.0874	463.0883, 300.0232, 271.0223, 255.0302	C_21_H_20_O_12_	Isoquercitrin	1.34%	1.94%	394.00 ± 1.00	302.50 ± 7.68
6	13.10	594	593.1496	285.03880, 517.0558	C_27_H_30_O_15_	Kaempferol-3-*O*-rutinoside	1.93%	/	>1000	337.85 ± 10.53
7	13.10	434	433.0774	433.0413, 300.9984, 146.9651	C_20_H_18_O_11_	Avicularin	/	1.60%	>1000	420.91 ± 8.86
8	13.80	448	447.0894	447.0874, 285.0426	C_21_H_20_O_11_	Astragalin	/	/	506.67 ± 91.39	159.75 ± 11.02
9	14.60	418	417.0823	417.0823, 284.0317, 146.9645	C_20_H_18_O_10_	Kaempferol-3-*O*-arabinoside	5.42%	/	819.67 ± 49.08	390.11 ± 22.21
10	17.50	302	301.0347	301.0360, 178.9953, 151.0042	C_15_H_10_O_7_	Quercetin	14.88%	8.88%	258.67 ± 10.79	230.88 ± 7.83
11	17.70	286	285.0426	285.0426	C_15_H_10_O_6_	Kaempferol	/	/	293.33 ± 48.81	422.21 ± 4.45
Flavan-3-ols										
12	5.29	290	289.0706	289.0724, 245.0795, 203.0677	C_15_H_14_O_6_	Catechin	4.96%	1.73%	100.67 ± 5.51	309.80 ± 14.11
13	6.50	290	289.0702	289.0724, 245.0795, 203.0677, 179.0330	C_15_H_14_O_6_	Epicatechin	1.69%	2.42%	94.33 ± 5.51	356.79 ± 13.65
Procyanidins										
14	5.78	578	577.1343	577.1353, 407.0775, 289.0719	C_30_H_26_O_12_	Procyanidin B_2_	2.05%	/	>1000	>500
15	6.18	866	865.1999	865.1999, 677.1541, 577.1389, 289.0734	C_45_H_38_O_18_	Procyanidin C_3_	8.85%	/	78.67 ± 2.31	46.98 ± 4.43
Phenolic acids										
16	5.40	354	353.0870	353.0843, 191.0537	C_16_H_18_O_9_	Chlorogenic acid	/	/	826.67 ± 22.72	343.87 ± 12.27
17	6.02	180	179.0331	179.0300, 135.0451	C_9_H_8_O_4_	Caffeic acid	2.86%	/	387.33 ± 34.08	143.25 ± 1.70
Ellagic acid and derivatives										
18	9.77	434	433.0405	433.070, 300.0276, 271.0250	C_19_H_14_O_12_	Ellagic acid pentoside	1.31%	/	/	/
19	10.90	302	300.9972	300.9999, 229.0135, 160.8405	C_14_H_6_O_8_	Ellagic acid	3.84%	/	>1000	262.43 ± 19.61

**Table 3:** Molecular docking analysis of active compounds in the ethyl acetate fraction of raspberry stems and leaves.

NO.	Compounds	Molecular Docking (α-Amylase)			Molecular Docking (α-Glucosidase)		
		Affinity Energy (kcal/mol)	n(H-Bond)	Active Amino Acid Residues	Affinity Energy (kcal/mol)	n(H-Bond)	Active Amino Acid Residues
Positive Control							
	Acarbose	−9.09	15	LYS-156, TYR-158, SER-240, ASP-242, GLN-279, HIS-280, ASP-307, PRO-312, LEU-313, ARG-315, ASP-352, GLN-353, GLU-411, ASN-415, ARG-442	−9.08	18	TRP-59, GLN-63, TYR-151, VAL-163, LYS-200, HIS-201, GLU-233, GLU-240, ASP-300, GLY-306
Flavonols							
1	Quercetin-3-*O*-β-D-glucosyl-7-*O*-β-D-gentiobioside	−9.55	11	TRP-59, GLN-63, HIS-101, TYR-151, ARG-195, ASP-197, LYS-200, HIS-201, GLU-233, ILE-235, HIS-299, ASP-300	−10.36	21	TYR-158, SER-240, SER-241, ASP-242, ALA-281, ASN-302, SER-304, ASP-307, THR-310, PRO-312, ARG-315, GLU-332, GLU-411
2	Rutin	−8.05	10	TYR-62, TYR-151, ALA-198, LYS-200, HIS-201, GLU-233, GLU-240, HIS-305, GLY-306	−9.56	22	LYS-156, TYR-158, ASP-233, SER-240, GLU-277, GLN-279, HIS-280, ARG-315, ASP-352, ARG-442
3	Hyperoside	−7.39	8	TRP-59, TYR-62, TYR-151, ARG-195, GLU-233, ILE-235, HIS-299, HIS-305	−7.90	12	LYS-156, TYR-158, SER-240, ASP-242, THR-306, ASP-307, SER-311, PRO-312, LEU-313, PHE-314, ARG-315, ASN-415
4	Quercetin-3-glucuronide	−7.13	7	HIS-101, ASP-197, LYS-200, HIS-201, ASP-300	−8.08	12	LYS-156, TYR-15, SER-240, SER-241, ASP-242, HIS-280, ASP-307, THR-310, PRO-312, LEU-313, GLU-411, ASN-415
5	Isoquercitrin	−6.94	10	HIS-101, ASP-197, LYS-200, HIS-201, GLU-233, HIS-299, ASP-300, HIS-305	−8.15	24	TYR-158, GLY-160, GLU-277, GLN-279, HIS-280, ASP-307, THR-310, SER-311, PRO-312, ASP-352, GLU-411, ASN-415, ARG-442
6	Kaempferol-3-*O*-rutinoside	−8.76	10	TRP-59, GLN-63, HIS-101, ASP-197, HIS-201, GLU-233, HIS-299, ASP-300, HIS-305	−7.19	4	ARG-329, GLU-204, GLU-203
Flavonols							
7	Avicularin	−7.24	11	TRP-59, TYR-62, TYR-151, ARG-195, ASP-197, GLU-233, ILE-235, HIS-299, ASP-300, HIS-305, GLY-306	−7.57	13	LYS-156, SER-240, SER-241, ASP-242, HIS-280, ASP-307, THR-310, PRO-312, LEU-313, PHE-314, ARG-315, GLU-411, ASN-415
8	Astragalin	−6.91	7	TYR-151, GLU-233, ILE-235, HIS-299	−7.92	14	TYR-58, GLY-160, GLN-279, HIS-280, THR-306, ASP-307, SER-311, PRO-312, ARG-315, ASP-352, GLN-353, GLU-411, ASN-415, ARG-442
9	Kaempferol-3-*O*-arabinoside	−7.14	17	TRP-59, GLN-63, ARG-195, ASP-197, ALA-198, GLU-233, HIS-299, ASP-300, GLY-306	−7.60	11	LYS-156, SER-241, ASP-242, HIS-280, ASP-307, SER-311, GLU-411, ASN-415
10	Quercetin	−5.79	8	TYR-62, HIS-101, ASP-197, LYS-200, HIS-201, ILE-235	−5.85	7	TYR-158, HIS-280, ASP-307, THR-310, SER-311, PRO-312, GLU-411
11	Kaempferol	−5.64	8	GLN-63, HIS-201, GLU-233, HIS-299, ASP-300	−6.06	5	TYR-158, GLU-277, ARG-315, GLN-353, ARG-442
Flavan-3-ols							
12	Catechin	−5.71	10	TRP-59, GLN-63, TYR-151, HIS-201, GLU-233, ASP-300, HIS-305, GLY-306	−6.09	14	LYS-156, THR-310, SER-311, PRO-312, LEU-313, ARG-315, GLU-411, ASN-415
13	Epicatechin	−6.15	12	TYR-151, LYS-200, HIS-201, ILE-235, ASP-300	−6.20	7	SER-157, TYR-158, SER-240, SER-241, ASP-242, ASP-307, ARG-315
Procyanidins							
14	Procyanidin B_2_	−7.41	14	TRP-59, GLN-63, TYR-151, HIS-201, HIS-299, ASP-300, HIS-305, GLY-306	−8.68	15	LYS-156, SER-157, TYR-158, GLY-160, SER-240, SER-241, ASP-242, GLN-279, ASP-307, PRO-312, LEU-313, PHE-314, ASP-352, GLU-411, ARG-442
15	Procyanidin C_3_	−9.33	24	TRP-59, GLN-63, TYR-151, VAL-163, LYS-200, GLU-233, GLU-240, ASP-300, HIS-305, GLY-306, ASP-356	−10.45	14	SER-157, TYR-158, SER-240, ASP-242, PRO-243, LEU-246, ASN-247, HIS-280, THR-310, PRO-312, LEU-313, PHE-314, ARG-315, GLU-411
Phenolic acids							
16	Chlorogenic acid	−6.99	9	TYR-62, HIS-101, ASP-197, LYS-200, GLU-233, ILE-235	−6.77	9	TYR-158, SER-240, ASP-242, THR-306, LEU-313, ARG-315, ASN-350, ASP-352, GLN-353
17	Caffeic acid	−4.69	8	ASP-197, ASP-300, HIS-305, GLY-306	−5.31	6	LYS-156, TYR-158, SER-240, SER-241, ASP-242, LEU-313
Ellagic acid and derivatives							
18	Ellagic acid	−5.42	6	TRP-59, TYR-151, HIS-201, GLU-233, HIS-299	−5.91	8	LYS-156, TYR-158, GLN-239, SER-240, SER-241, ASP-242, GLN-279, HIS-280

## Data Availability

Data Availability Statement: The original contributions presented in this study are included in the article/Supplementary Materials. Further inquiries can be directed to the corresponding author.

## Supplementary Materials

The following supporting information can be downloaded at: https://www.mdpi.com/article/10.3390/foods15071134/s1.

## Abbreviations

The following abbreviations are used in this manuscript:

UF-LC/MS	Ultrafiltration–Liquid Chromatography–Mass Spectrometry
UPLC-MS/MS	Ultra-Perfomance Liquid Chromatography–Mass Spectrometry/Mass Spectrometry
HPLC	High-Performance Liquid Chromatography
TLC	Thin-Layer Chromatography
LC-MS/MS	Liquid Chromatography–Mass Spectrometry/ Mass Spectrometry
ESI	Electrospray ionization
NMR	Nuclear Magnetic Resonance
r/min	Revolutions Per Minute
DMSO	Dimethyl sulfoxide
DNS	3,5-Dinitrosalicylic acid
UPLC-PDA-MS (TOF)	Ultra-high-Performance Liquid Chromatography–Mass Spectrometry with Time-of-Flight Detection
