# Supplementary material for: Discovery of Inhibitory Active Ingredients for α-Amylase and α-Glucosidase from Raspberry (Rubus idaeus L.) Stems and Leaves Guided by Affinity Ultrafiltration and UPLC-QTOF-MS/MS
Source: Foods. 2026 Mar 25;15(7):1134. doi: 10.3390/foods15071134 (PMC13072966; doi:10.3390/foods15071134)
Supplement: Supplementary file 1 [file foods-15-01134-s001.zip › foods-4189888-supplementary.pdf]

Supplementary Materials

2.2 Extraction

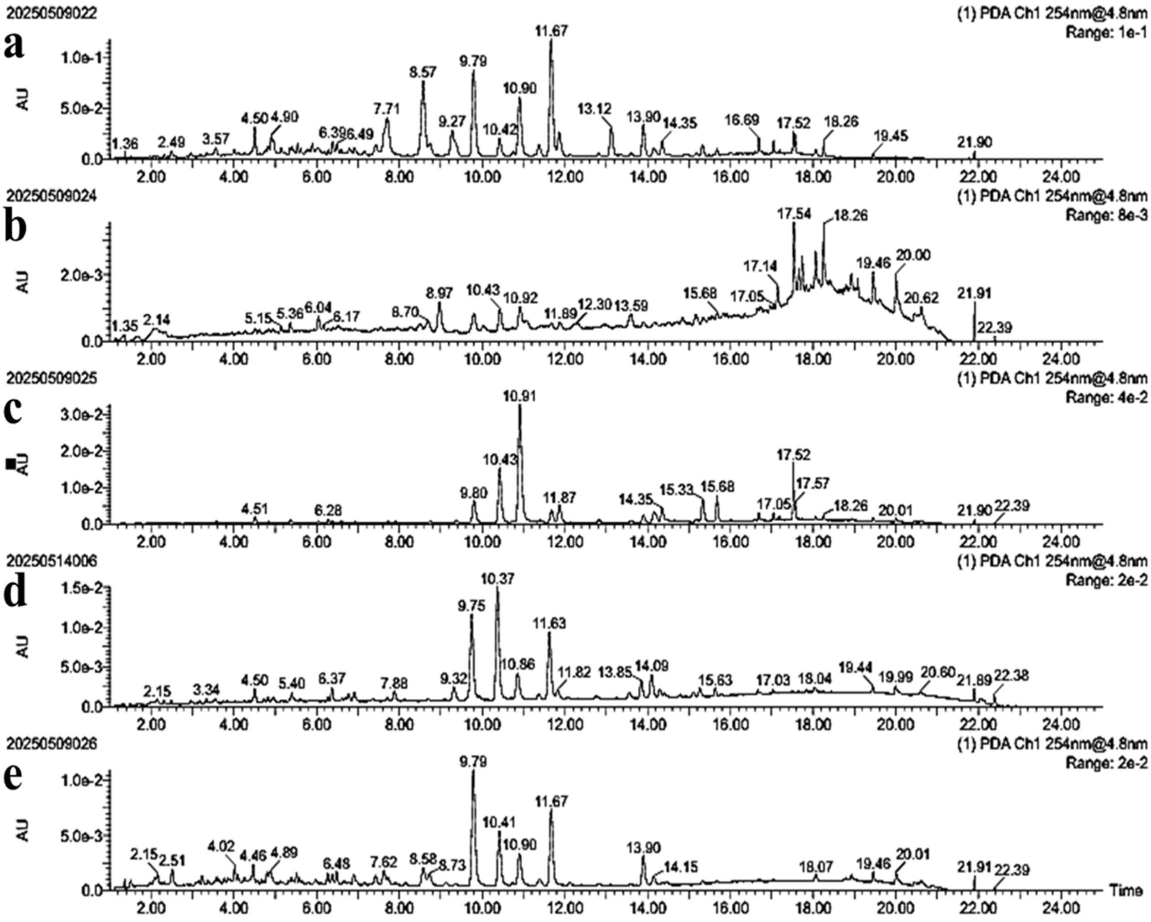

Figure S1. PDA spectra of raspberry stem and leaf extracts (a) and four extraction fractions: dichloromethane fraction (b), ethyl acetate fraction (c), *n*-butanol fraction (d), aqueous fraction (e).

2.4 Analysis activity of α-glucosidase

Table S1. Mass spectrometry parameters for three sugars.

Compounds	RT min	Mw	Mode	Parent ion m/z	Product ion m/z	Cone voltage /V	Collision energy /V
Maltose	1.34	286.2	ESI^-	340.8	160.8*	58	6
					178.8	58	8
Glucose	0.97	302.0	ESI^-	178.8	88.9*	36	8
					118.9	36	10
Xylitol	0.85	316.2	ESI^-	150.8	88.9*	6	12
					101.0	6	12

2.4.1 Method validation for α-glucosidase inhibition assay

The α-glucosidase inhibition assay was performed using maltose as substrate, and the generated glucose was quantified by LC-ESI-MS/MS with xylitol as internal standard. The method was validated for both glucose and maltose to ensure reliability.

Maltose as substrate: By measuring maltose at different concentrations, the intra day precision, inter day precision, and accuracy of maltose were tested. The maltose concentration was calculated by using the obtained calibration curve. The precision of each concentration was represented by relative standard deviation (RSD), and the accuracy was represented by relative error (RE). Precision and accuracy: RSD was within 15%, and accuracy did not exceed 15% of the actual sample value. The results were shown in Table S2. The stability and reliability of maltose measurement were also validated. The linearity of maltose quantification was established over 0.05-10 μg/mL (y = 72.1289x + 90.5583, R^2= 0.9990), with an LOD of 0.3 μg/mL, LOQ of 1.0 μg/mL.

Table S2. Results of precision and accuracy of methodology investigation.

Concentration (μg/mL)	Mean concentration (μg/mL)	Relative error （%）	Precision （%）	
		RE	Intra-day precision RSD	Iner-day precision RSD
7.0	7.141 0.06	13.09%	0.79%	7.89%
2.5	2.999 0.01	0.06%		
0.3	0.313 0.01	1.34%		

Table S3. Recovery rate of added sample (n=3).

Component	Content of mixed standard/μg	Addition/μg	Measured quantity/μg	Average recovery rate /%	RSD/ %
maltose	5162.3	5000	10756.0	104.28	7.81
	5342.6	5000	10607.3		
	4958.9	5000	9743.1		

These validation results confirm that the LC-MS/MS method is reliable, sensitive, and reproducible for quantifying both substrate stability and enzymatic glucose production in the α-glucosidase inhibition assay.

3.2. Analysis of affinity ultrafiltration

Table S4. *Metabolites identified in the raspberry stem and leaf extracts of byUPLC-QTOF-MS/MS in*

ESI$^-$ Modes.

NO.	Compounds	RT	[M-H]$^-$ (m/z)	Molecular formula	Mass error (ppm)	Reference
**1**	Quercetin-3-*O*-β-D-glucosyl-7-*O*-β-D-gentiobioside	5.01	787.1406	$C_{33}H_{40}O_{22}$	-4.55	standard
**2**	Catechin	5.29	289.0706	$C_{15}H_{14}O_6$	-2.13	standard
**3**	Chlorogenic acid	5.40	353.0870	$C_{16}H_{18}O_9$	0.74	standard
**4**	Procyanidin B_2	5.78	577.1343	$C_{30}H_{26}O_{12}$	0.53	standard
**5**	Caffeic acid	6.02	179.0331	$C_9H_8O_4$	-5.42	standard
**6**	Procyanidin C_3	6.18	865.1999	$C_{45}H_{38}O_{18}$	2.2	literature
**7**	Epicatechin	6.50	289.0702	$C_{15}H_{14}O_6$	-3.51	standard
**8**	Ellagic acid pentoside	9.77	433.0405	$C_{19}H_{14}O_{12}$	-0.05	literature
**9**	Ellagic acid	10.90	300.9972	$C_{14}H_6O_8$	-4.14	standard
**10**	Rutin	11.10	609.1487	$C_{27}H_{30}O_{16}$	6.13	standard
**11**	Hyperoside	11.40	463.0875	$C_{21}H_{20}O_{12}$	0.65	standard
**12**	Quercetin-3-glucuronide	11.60	477.0671	$C_{21}H_{18}O_{13}$	1.43	standard
**13**	Isoquercitrin	11.80	463.0874	$C_{21}H_{20}O_{12}$	0.43	standard
**14**	Kaempferol-3-*O*-rutinoside	13.10	593.1496	$C_{27}H_{30}O_{15}$	-0.67	standard
**15**	Avicularin	13.10	433.0774	$C_{20}H_{18}O_{11}$	0.72	standard
**16**	Astragalin	13.80	447.0894	$C_{21}H_{20}O_{11}$	-6.26	standard
**17**	Kaempferol-3-*O*-arabinoside	14.60	417.0823	$C_{20}H_{18}O_{10}$	0.25	standard
**18**	Quercetin	17.50	301.0347	$C_{15}H_{10}O_7$	0.01	standard
**19**	Kaempferol	17.70	285.0426	$C_{15}H_{10}O_6$	3.92	standard

3.3. Isolation and Identification of Compounds

The HRMS and NMR information for the isolated compounds (1-13, 14):

Compound **1**: $C_{15}H_{10}O_6$, yellow powder, ESI-MS $[M-H]^-$ m/z: 285.0426, ^{1}H-NMR (MeOD-d4) *δ*: 6.20 (1H, d, *J* = 2.1 Hz, H-6), 6.41 (1H, d, *J* = 2.1 Hz, H-8), 8.10 (2H, d, *J* = 8.0 Hz, H-2′, H-6′), 6.92 (2H, d, *J* = 8.0 Hz, H-3′, H-5′); ^{13}C-NMR (400Hz, MeOD): 146.65 (C-2), 135.74 (C-3), 175.98 (C-4), 156.86 (C-5), 97.85 (C-6), 164.17 (C-7), 93.05 (C-8), 161.13 (C-9), 103.15 (C-10), 122.33(C-1′), 129.28(d, C-2′, 6′), 114.90 (d, C-3′, 5′), 159.16 (C-4′). Based on literature comparison and high-resolution mass spectrometry analysis, the data are consistent with previous studies . Therefore, compound **1** was identified as **kaempferol**.

Compound **2**: $C_{14}H_6O_8$, yellow powder, ESI-MS $[M-H]^-$ m/z: 300.9999, ^{1}H NMR (DMSO-d6) *δ*: 7.39 ppm (2H, s, H-4,, H-9); ^{13}C NMR (400 MHz, DMSO): 112.80 (d, C-1, C-1′), 108.09 (d, C-2, C-2′), 110.69 (d, C-3, C-3′), 148.58 (d, C-4, C-4′), 140.07 (d, C-5, C-5′), 136.85 (d, C-6, C-6′), 159.61 (d, C-7, C-7′). Based on literature comparison and high-resolution mass spectrometry analysis, the data are consistent with previous studies. Therefore, compound **2** was identified as **ellagic acid** .

Compound **3**: $C_{27}H_{30}O_{16}$, yellow powder, ESI-MS $[M-H]^-$ m/z: 609.1487 $[M-H]^-$; ^{1}H NMR (MeOD-d4) *δ*: 7.57 (1H, d, *J* = 2.1 Hz, H-2), 7.53 (1H, dd, *J* = 2.2, 8.4 Hz, H-6), 6.78 (1H, d, *J* = 8.4 Hz, H-5), 6.30 (1H, d, *J* = 2.1 Hz, H-8), 6.11 (1H, d, *J* = 2.1 Hz, H-6), 5.01 (1H, d, *J* = 7.6 Hz, H-1"), 4.42 (1H, d, *J* = 1.6 Hz, H-1"), 3.72 - 3.53 (peaks corresponding to overlapping disaccharide units), 1.02 (3H, d, *J* = 6.2 Hz, H-6); ^{13}C NMR (400 MHz, MeOD): 157.94 (C-2), 134.22 (C-3), 178.02 (C-4), 161.59 (C-5), 98.56 (C-6), 164.68 (C-7), 93.47 (C-8), 157.12 (C-9), 104.22 (C-10), 122.15 (C-1′), 114.66 (C-2′), 144.45 (C-3′), 148.42 (C-4′), 116.28 (C-5′), 121.72 (C-6′), 103.31 (C-1″), 74.33 (C-2″), 76.78 (C-3″), 69.99 (C-4″), 75.82 (C-5″), 67.15 (C-6″), 101.02 (C-1‴), 70.70 (C-2‴), 70.83 (C-3‴), 72.53 (C-4‴), 68.31 (C-5‴), 16.48 (C-6‴). Based on literature comparison and LC-MS analysis, the data are consistent with the reference literature. Therefore, the compound **3** was identified as **rutin**.

Compound **4**: $C_{21}H_{20}O_{12}$, yellow powder, ESI-MS $[M-H]^-$ m/z: 463.0875 $[M-H]^-$; ^{1}H-NMR (DMSO-d6) *δ*: 12.63 (1H, s, 5-OH), 6.20 (1H, d, *J* = 2.0 Hz, H-6), 6.41 (1H, d, *J* = 2.0 Hz, H-8), 7.53 (1 H, d, *J* = 2.2 Hz, H-2′), 6.82 (1H, d, *J* = 8.5 Hz, H-5 ′), 7.67 (1H, dd, *J* = 8.5, 2.2 Hz, H-6′), 5.38 (1H, d, *J* = 7.6 Hz, H-1″); ^{13}C NMR (400 MHz, DMSO): 102.15 (gal C-1), 71.66 (gal C-2), 73.64 (gal C-3), 68.38 (gal C-4), 76.31 (gal C-5), 60.60 (gal C-6), 121.55 (C-1′), 115.65 (C-2′), 145.30 (C-3′), 148.93 (C-4′), 116.40 (C-5′), 122.47 (C-6′), 156.77 (C-2), 133.94 (C-3), 177.95 (C-4), 161.69 (C-5), 164.62 (C-7), 99.14 (C-6), 93.97 (C-8), 156.69 (C-9), 104.37 (C-10). Based on literature comparison and high-resolution mass spectrometry analysis, the data are consistent with previous studies. Therefore, compound **4** was identified as **hyperoside**.

Compound **5**: $C_{21}H_{20}O_{12}$, yellow powder, ESI-MS $[M-H]^-$ m/z: 463.0874 $[M-H]^-$; ^{1}H-NMR (DMSO-d6) *δ*: 12.58 (1H, s, 5-OH), 7.59 (d, *J* = 2.2 Hz, 1H, H-6′), 7.56 (dd, *J* = 8.4, 2.3 Hz, 1H, H-2′), 6.84 (d, *J* = 8.4 Hz, 1H, H-5′), 6.39 (d, *J* = 2.1 Hz, 1H, H-8), 6.19 (d, *J* = 2.1 H, 1H, H-6), 5.44 (d, *J* = 7.3 Hz, 1H, H-1″); ^{13}C NMR (400 MHz, DMSO): 157.51 (C-2), 134.66 (C-3), 178.70 (C-4), 162.30 (C-5), 99.65 (C-6), 165.17 (C-7), 94.59 (C-8), 157.63 (C-9), 105.23 (C-10), 122.79 (C-1′), 116.23 (C-2′), 149.40 (C-3′), 145.76 (C-4′), 117.32 (C-5′), 122.51 (C-6′), 102.29 (C-1″), 75.21 (C-2″), 77.56 (C-3″), 70.97 (C-4″), 78.56 (C-5″), 62.06 (C-6″). Based on literature comparison and high-resolution mass spectrometry analysis, the data are consistent with previous studies. Therefore, compound **5** was identified as **isoquercitrin**.

Compound **6**: $C_{15}H_{14}O_6$, white crystalline solid, ESI-MS $[M-H]^-$ m/z: 289.0702; ^{1}H-NMR (MeOD-d4) δ: 2.81 (1H, dd, *J* = 2.8 Hz, 2.9 Hz, H-4), 2.90 (1H, dd, *J* = 4.0 Hz, 4.5 Hz, H-4), 4.22 (1H, s, H-3), 4.86 (1H, s, H-2), 5.98 (1H, d, *J* = 2.2 Hz, H-6), 6.00 (1H, d, *J* = 2.3 Hz, H-8), 6.82 (2H, s, H-5', H-6'), 7.00 (1H, s, H-2'); ^{13}C NMR (400MHz, MeOD): 78.48 (C-2), 66.10 (C-3), 27.87 (C-4), 156.18 (C-5), 95.01 (C-6), 156.54 (C-7), 94.51 (C-8), 155.92 (C-9), 98.69 (C-10), 130.90 (C-1'), 113.93 (C-2'), 144.38 (C-3'), 144.55 (C-4'), 118.02 (C-5'), 114.52 (C-6'). Based on literature comparison and high-resolution mass spectrometry analysis, the data are consistent with previous studies. Therefore, compound **6** was identified as **epicatechin**.

Compound **7**: $C_{15}H_{14}O_6$, yellow powder, ESI-MS $[M-H]^-$ m/z: 289.0706; ^{1}H-NMR (MeOD-d4) δ: 6.83 (1H, d, *J* = 1.9 Hz, H-2), 6.76 (1H, d, *J* = 8.0 Hz, H-5), 6.71 (1H, dd, *J* = 2.0, 8.4 Hz, H-6), 5.92 (1H, d, *J* = 2.4 Hz, H-8), 5.85 (1H, d, *J* = 2.4 Hz, H-6), 4.56 (1H, d, *J* = 7.6 Hz, H-2), 3.97 (1H, td, *J* = 7.9, 5.5 Hz, H-3), 2.85 (1H, dd, *J* = 5.6, 16.0 Hz, H-4), 2.50 (1H, dd, *J* = 8.0, 16.0 Hz, H-4); ^{13}C NMR (400 MHz, MeOD): 156.36 (C-7), 156.12 (C-5), 155.46 (C-9), 144.86 (C-3), 144.83 (C-4), 130.82 (C-1'), 118.64 (C-6), 114.68 (C-2'), 113.86 (C-5), 99.41 (C-10), 81.46 (C-2), 67.43 (C-3), 27.13 (C-4). Based on literature comparison and high-resolution mass spectrometry analysis, the data are consistent with previous studies. Therefore, compound **7** was identified as **catechin**.

Compound **8**: $C_{15}H_{10}O_7$, yellow powder, ESI-MS $[M-H]^-$ m/z: 301.0347; ^{1}H-NMR (MeOD-d4) δ: 6.11 (1H, d, *J* = 1.8 Hz, H-6), 6.32 (1H, d, *J* = 2.1 Hz, H-8), 6.81 (1H, d, *J* = 8.5 Hz, H-5'), 7.53 (1H, dd, *J* = 8.5 Hz, 2.1 Hz, H-6'), 7.63 (1H, d, *J* = 2.1 Hz, H-2'); ^{13}C NMR (400 MHz, MeOD): 165.42 (C-7), 162.31 (C-5), 158.11 (C-9), 148.70 (C-2), 146.11 (C-3′), 124.04 (C-1′), 121.75 (C-6′), 116.31 (C-5′), 116.02 (C-2′), 99.31 (C-6), 94.51 (C-8). Based on literature comparison and high-resolution mass spectrometry analysis, the data are consistent with previous studies. Therefore, compound **8** was identified as **quercetin**.

Compound **9**: $C_9H_8O_4$, yellow powder, ESI-MS $[M-H]^-$ m/z: 179.0331; ^{1}H-NMR (MeOD-d4) δ: 7.54 (1H, d, *J* = 15.9 Hz, H-7), 7.03 (1H, d, *J* = 2.1 Hz, H-2), 6.94 (H, dd, *J* = 8.2, 2.0 Hz, H-6), 6.77 (H, d, *J* = 8.2 Hz, H-5), 6.24 (1H, d, *J* = 15.8 Hz, H-8); ^{13}C NMR (400 MHz, MeOD): 127.68 (C-1), 114.83 (C-2), 146.92 (C-3), 149.56 (C-4), 116.47 (C-5), 122.91 (C-6), 146.80 (C-7), 115.10 (C-8), 169.77 (C-9). Based on literature comparison and high-resolution mass spectrometry analysis, the data are consistent with previous studies. Therefore, compound **9** was identified as **caffeic acid**.

Compound **10**: $C_{21}H_{20}O_{11}$, yellow powder, ESI-MS $[M-H]^-$ m/z: 447.0894; ^{1}H-NMR (MeOD-d4) δ: 12.62 (1H, s, H-5), 6.22 (1H, d, *J* = 1.9Hz, H-6), 6.45 (1H, d, *J* = 2.1 Hz, H-8), 8.05 (2H, d, *J* = 8.9 Hz, H-2′, H-6′), 6.90 (2H, d, *J* = 8.9 Hz, H3′-H5′), 10.33 (2H, s, H-7,H-4′), 5.47 (1H, d, *J* = 7.3 Hz, H-1″), 3.17 (2H, d, *J* = 8.9 Hz ,H-2″, H-3″), 3.14-3.06 (m, 2H, H-4″, H-5″), 3.57 (2H, d, *J* = 11.6 Hz, H-6″); ^{13}C NMR (400 MHz, MeOD): 156.64 (C-2), 134.48 (C-3), 177.67 (C-4), 161.20 (C-5), 98.83 (C-6), 164.26 (C-7), 93.91 (C-8), 156.46 (C-9), 104.23 (C-10), 121.18 (C-1′), 131.14 (C-2′, C-6′), 115.29 (C-3′, C-5′), 161.07 (C-4′), 101.08 (C-1″), 74.35 (C-2″), 76.51 (C-3″), 70.03 (C-4″) ,77.71 (C-5″), 60.97 (C-6″). Based on literature comparison and high-resolution mass spectrometry analysis, the data are consistent with previous studies. Therefore, compound **10** was identified as **astragalin**.

Compound **11**: $C_{27}H_{30}O_{15}$, yellow powder, ESI-MS $[M-H]^-$ m/z: 593.1496; ^{1}H-NMR (MeOD-d4) δ: 12.57 (1H, s, 5-OH), 7.99 (2H, d, *J* = 8.9 Hz, H-2', H-6'), 6.88 (2H, d, *J* = 8.9 Hz, H-3', H-5'), 6.42 (1H, d, *J* = 2.1 Hz, H-8), 6.21 (1H, d, *J* = 2.1 Hz, H-6), 5.32 (1H, d, *J* = 7.4 Hz,

H-1''), 5.09 (1H, dd, J = 9.3, 5.3 Hz, H-1'''), 3.44 - 3.17 (m, 10H); ^{13}C NMR (400 MHz, MeOD): 156.52 (C-2), 133.25 (C-3), 177.42 (C-4), 161.23 (C-5), 98.74 (C-6), 164.12 (C-7), 93.77 (C-8), 156.88 (C-9), 104.03 (C-10), 120.93 (C-1′), 130.92 (C-2′; C-6′), 115.11 (C-3′; C-5′), 159.90 (C-4′), 100.79 (C-1″), 74.20 (C-2″), 76.38 (C-3″), 70.61 (C-4″), 75.76 (C-5″), 66.92 (C-6″), 101.35 (C-1‴), 69.95 (C-2‴), 70.37 (C-3‴), 71.83 (C-4‴), 68.27 (C-5‴), 17.76 (C-6‴). Based on literature comparison and high-resolution mass spectrometry analysis, the data are consistent with previous studies, compound **11** was identified as **kaempferol-3-*O*-rutinoside**.

Compound **12**: $C_{20}H_{18}O_{10}$, yellow powder. ESI-MS m/z 417.104 [M-H]$^-$, RT: 14.55 min, fragments: 417.0823, 284.0317, 146.9645. By comparison with a reference standard, compound **12** was identified as **kaempferol-3-*O*-arabinoside**.

Compound **13**: $C_{21}H_{18}O_{13}$, white powder. ESI-MS m/z 477.0671 [M-H]$^-$, RT: 11.59 min, fragments: 451.3287, 301.0348, 178.997. By comparison with a reference standard, compound **13** was identified as **quercetin-3-*O*-glucuronide**.

Compound **15**: Molecular Formula: $C_{16}H_{18}O_9$, white powder. ESI- MS m/z 353.0870 [M-H]$^-$, retention time: 5.4 min, fragments: 353.0843, 191.0537. By comparison with a reference standard, compound **15** was identified as **chlorogenic acid**.

3.5 *Molecular docking*

Quercetin-3-O-β-D-glucosyl-7-O-β-D-gentiobioside

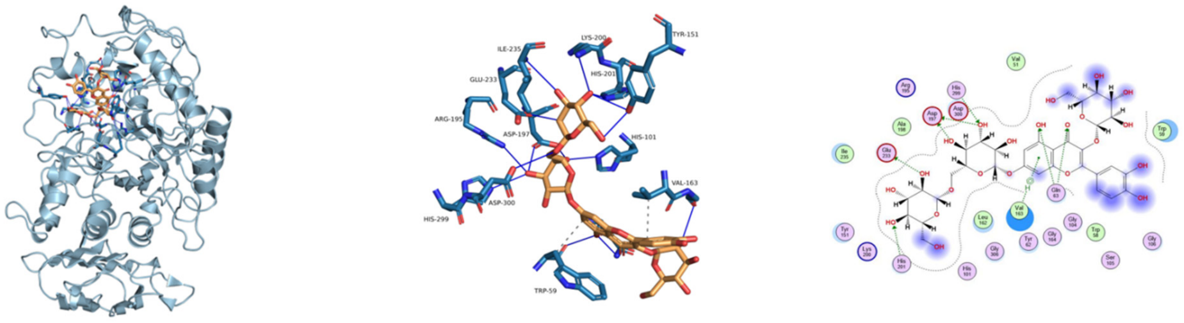

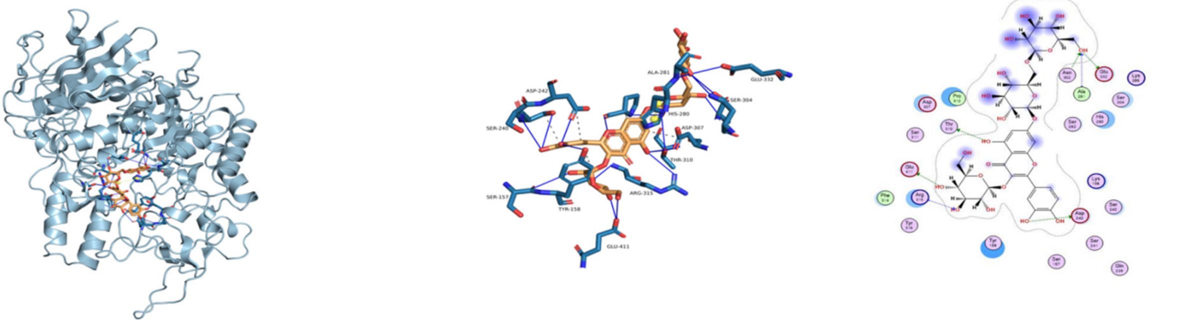

Rutin

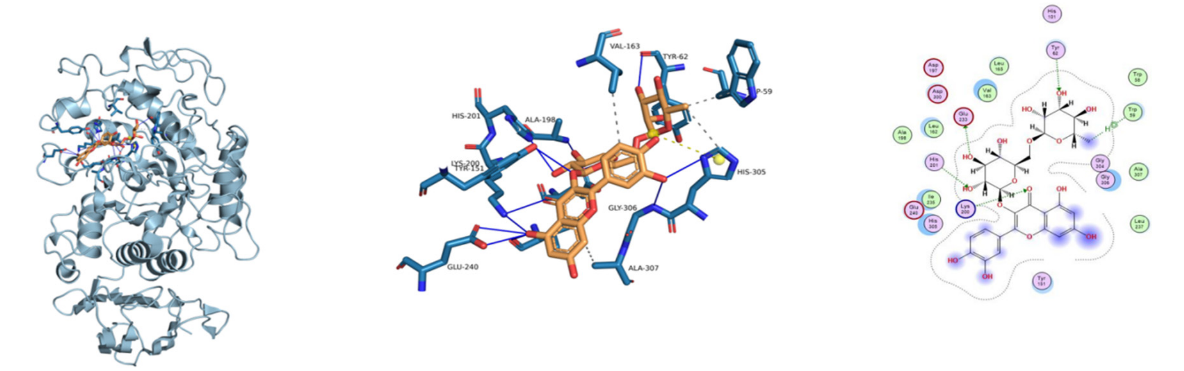

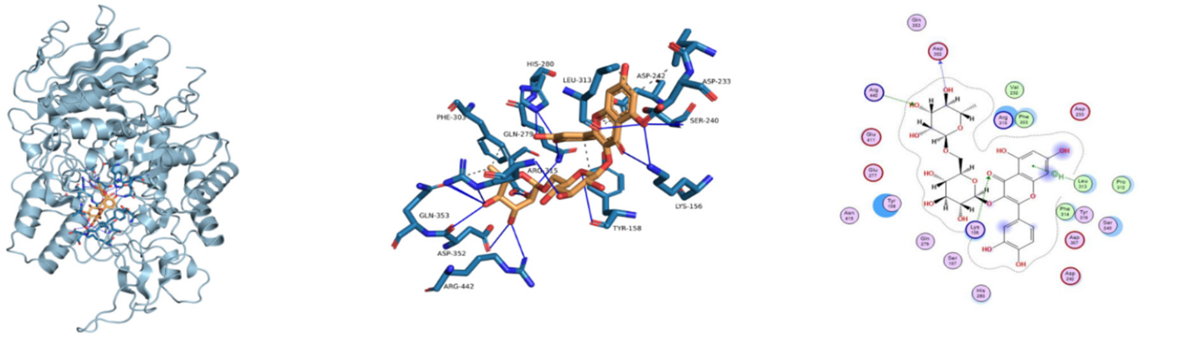

Hyperoside

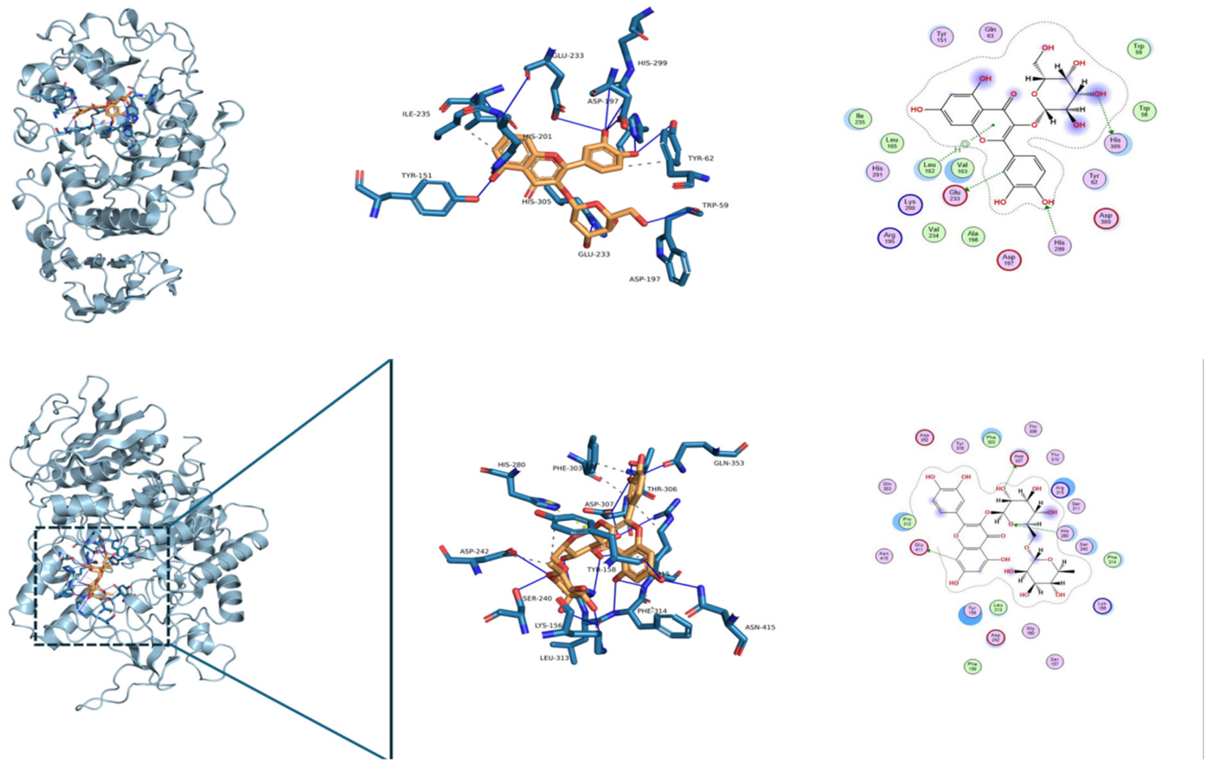

Quercetin-3-glucuronide

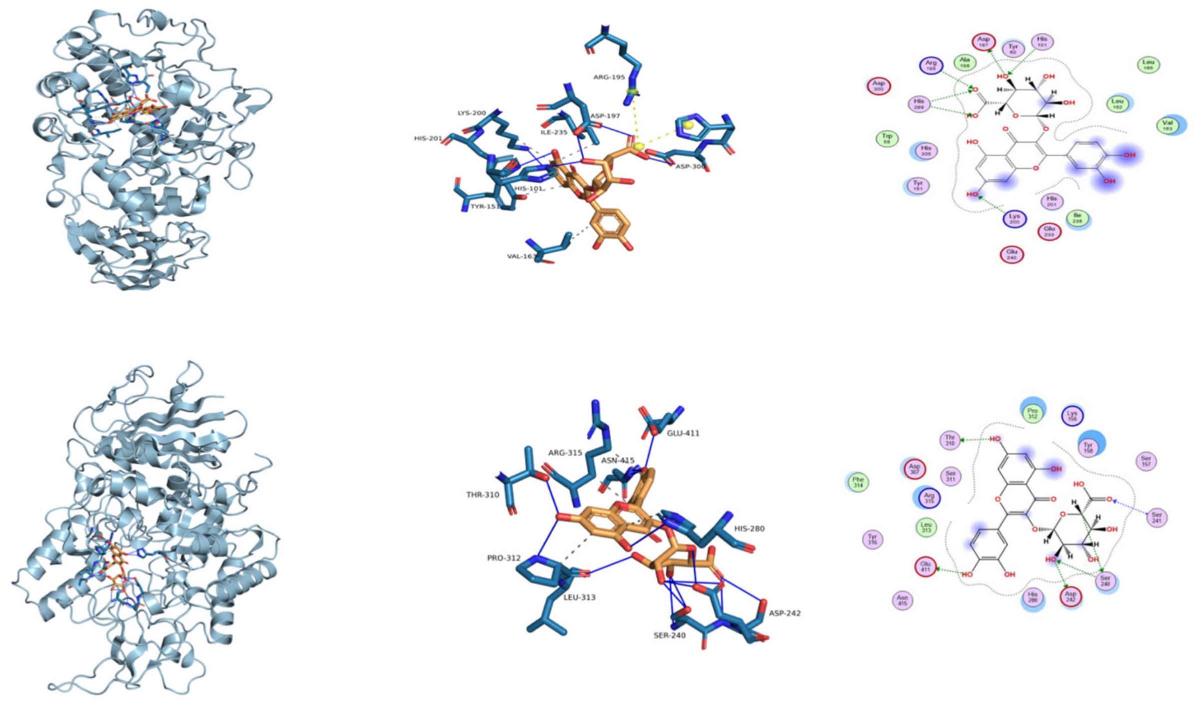

Isoquercitrin

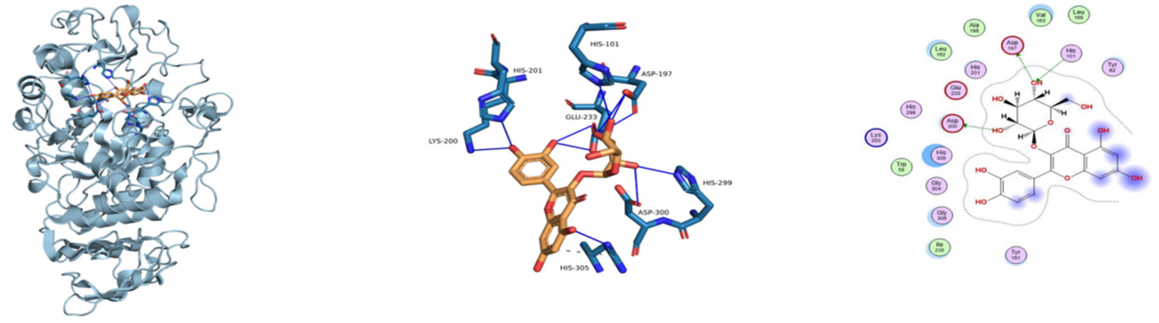

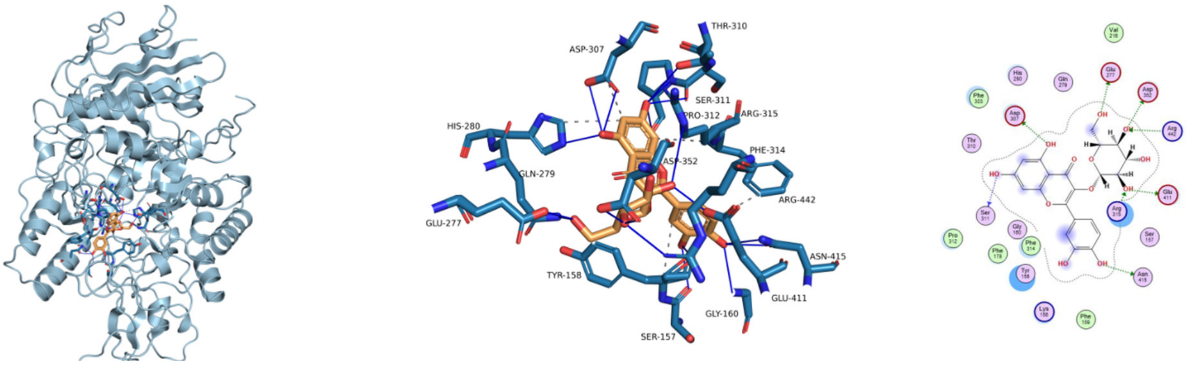

Kaempferol-3-O-rutinoside

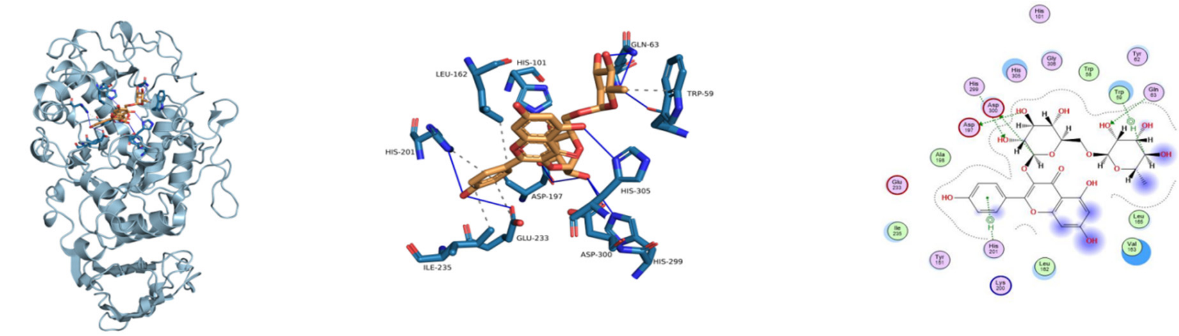

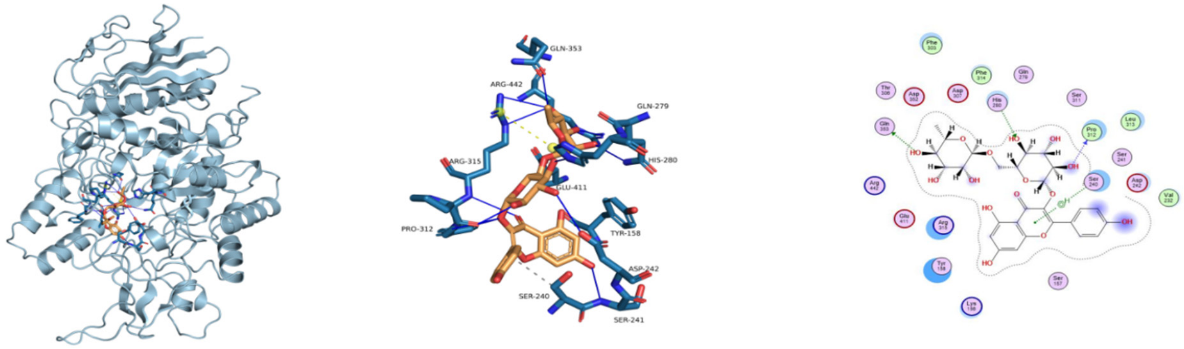

Avicularin

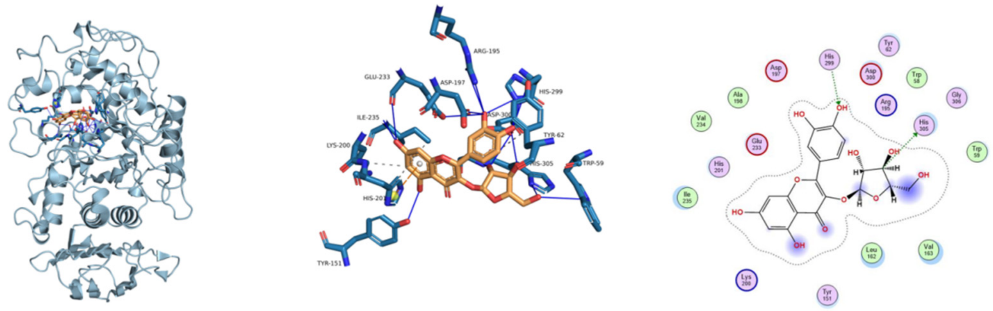

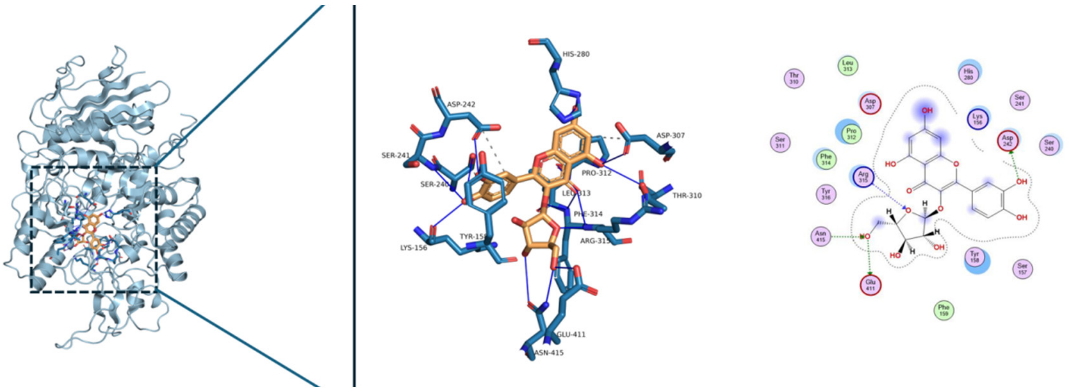

Astragalin

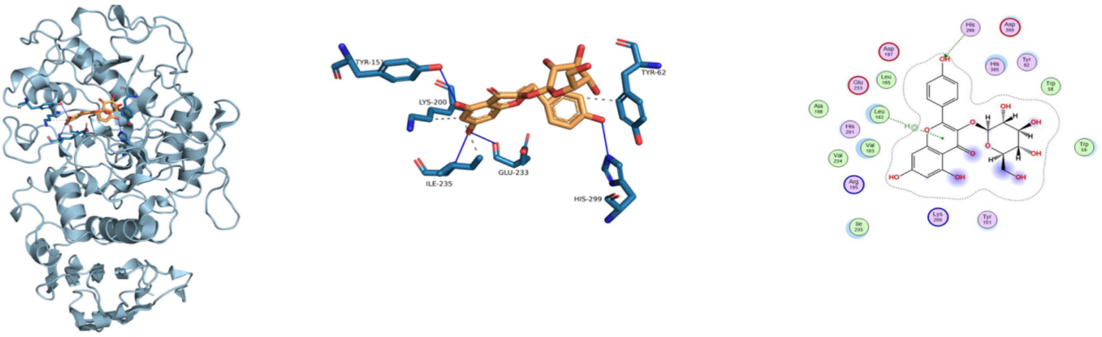

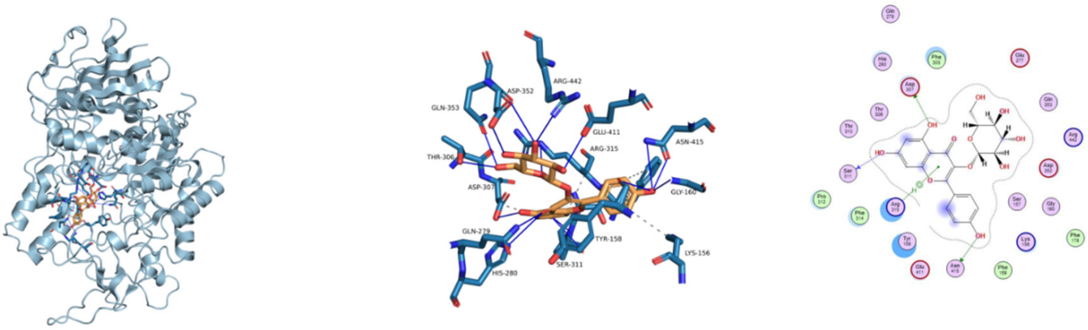

Kaempferol-3-O-arabinoside

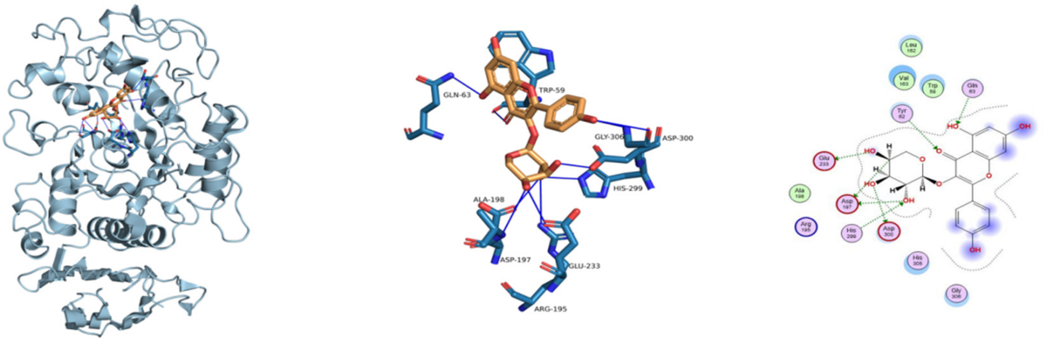

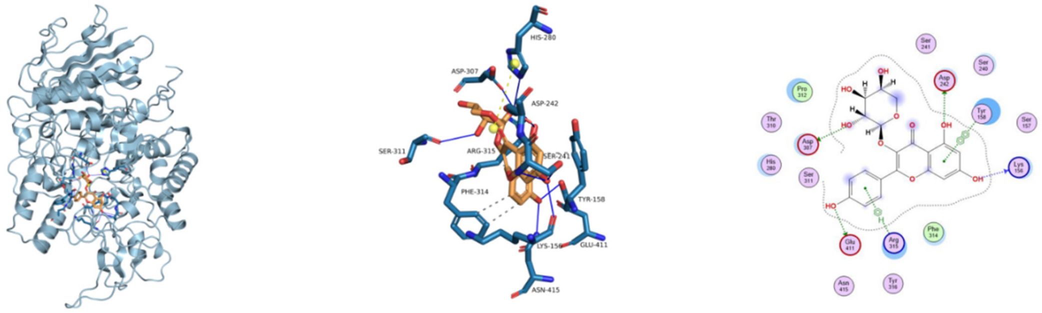

Quercetin

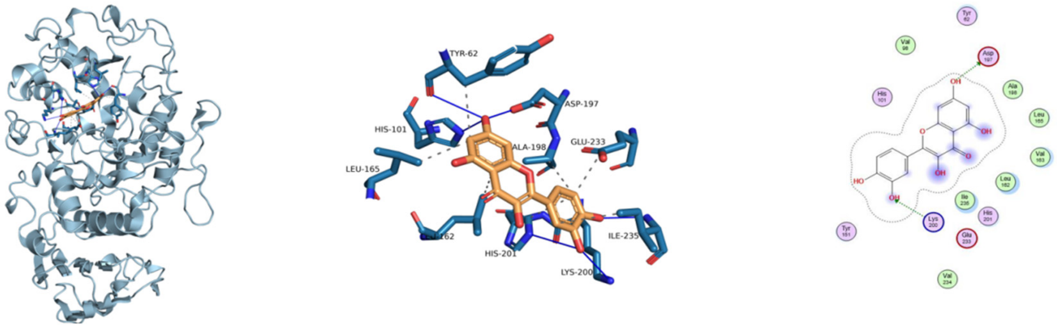

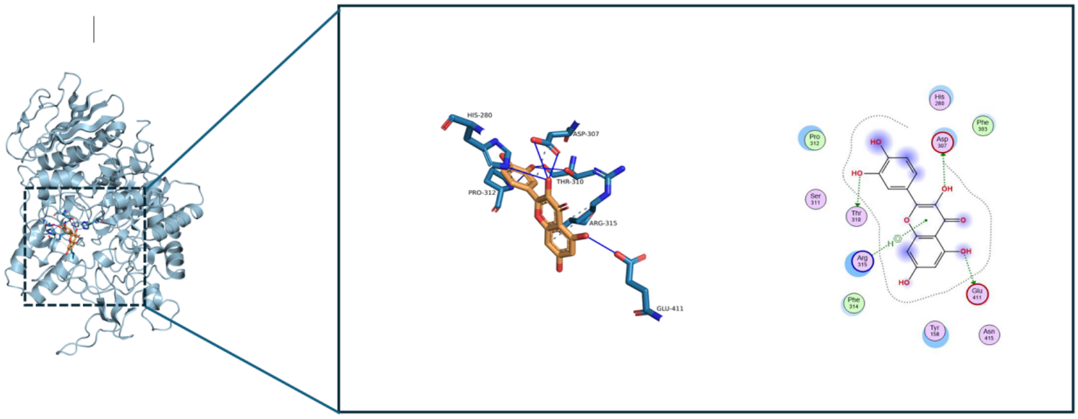

Kaempferol

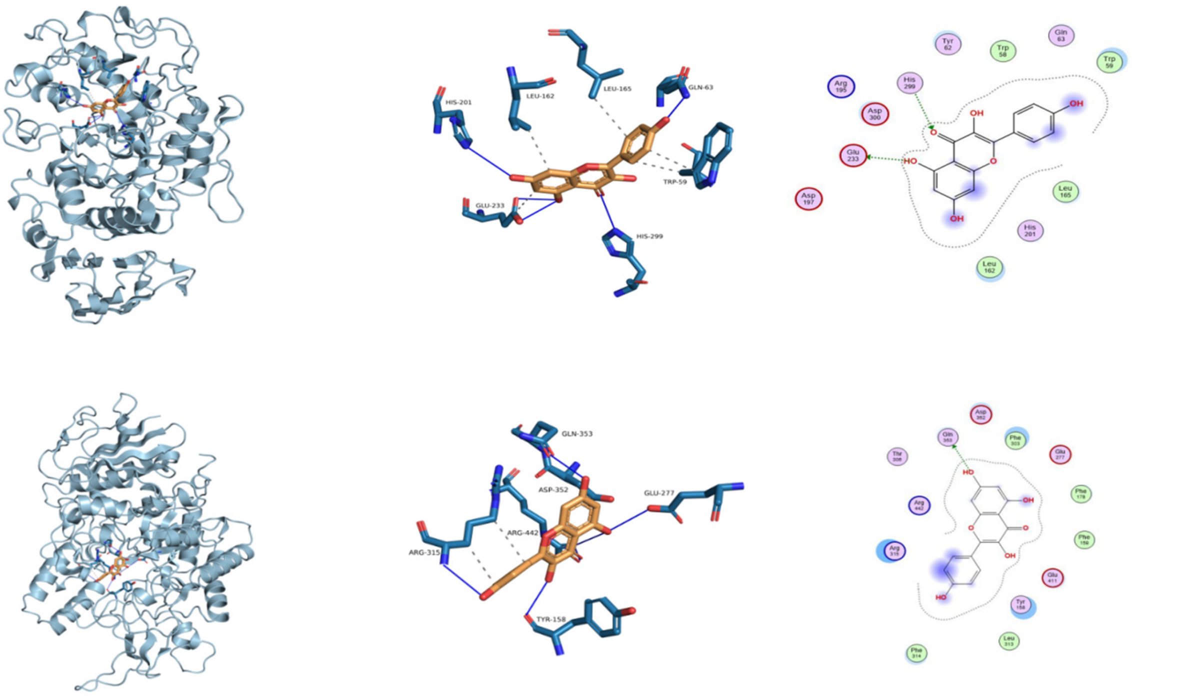

Catechin

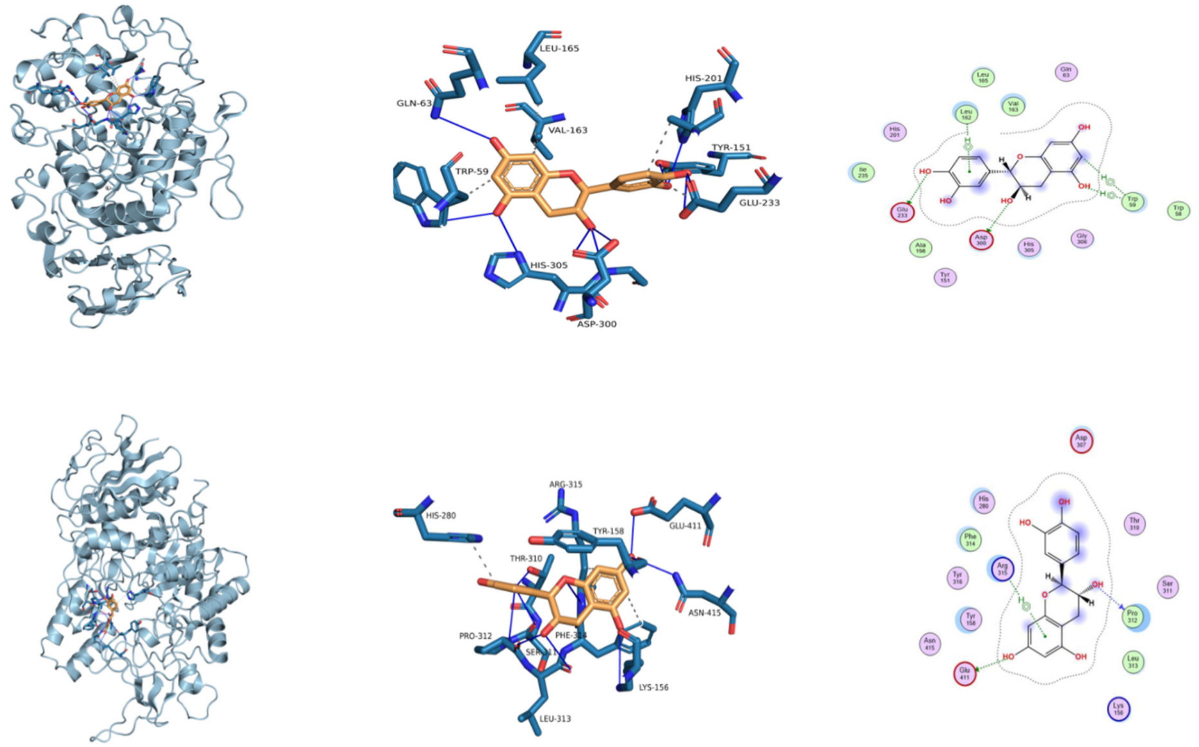

Epicatechin

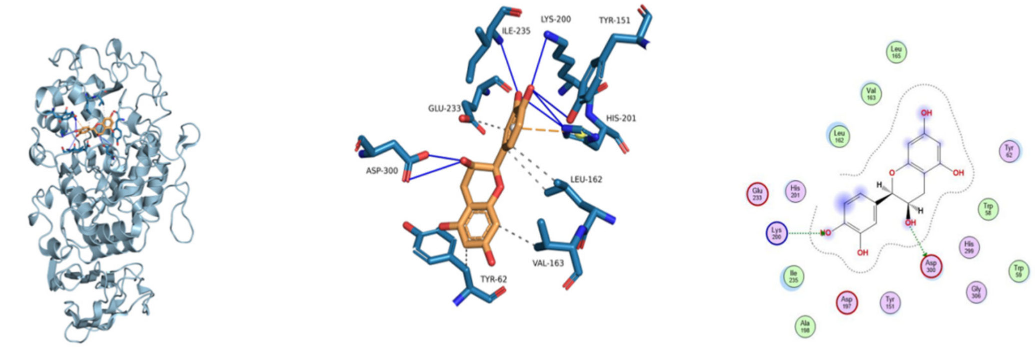

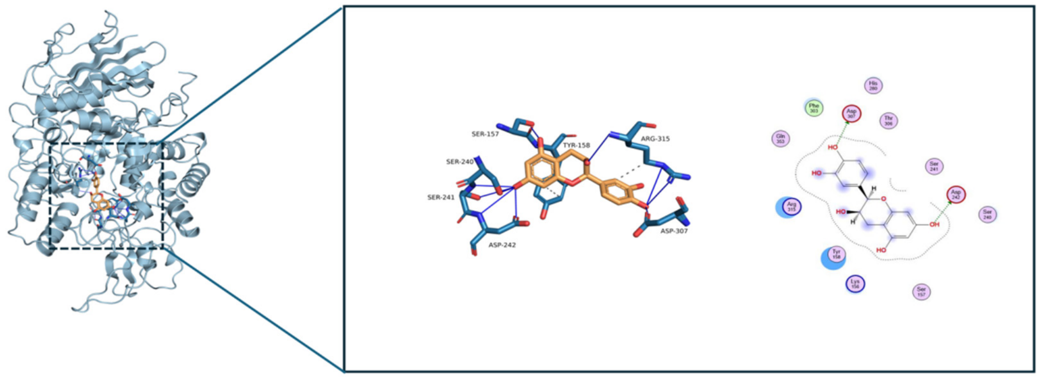

Procyanidin B2

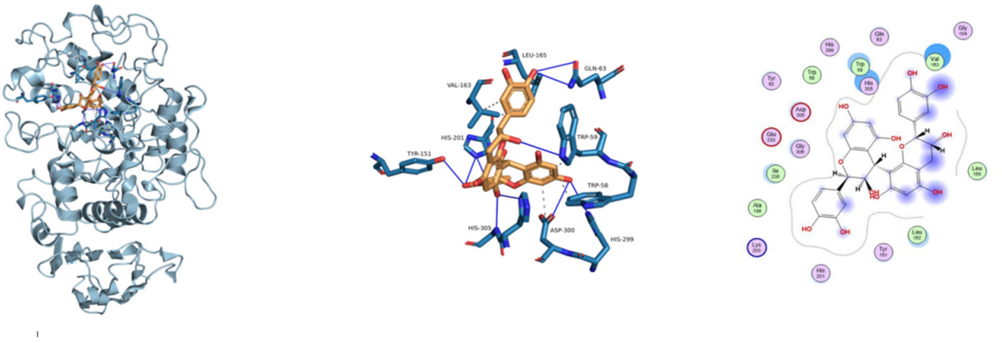

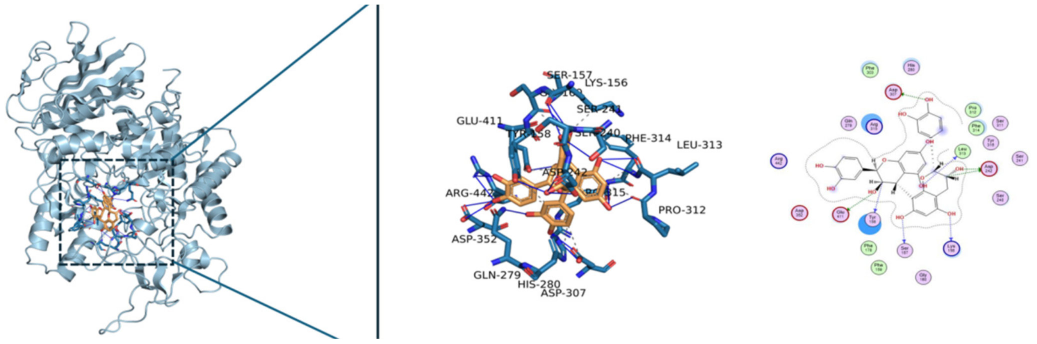

Procyanidin C_3

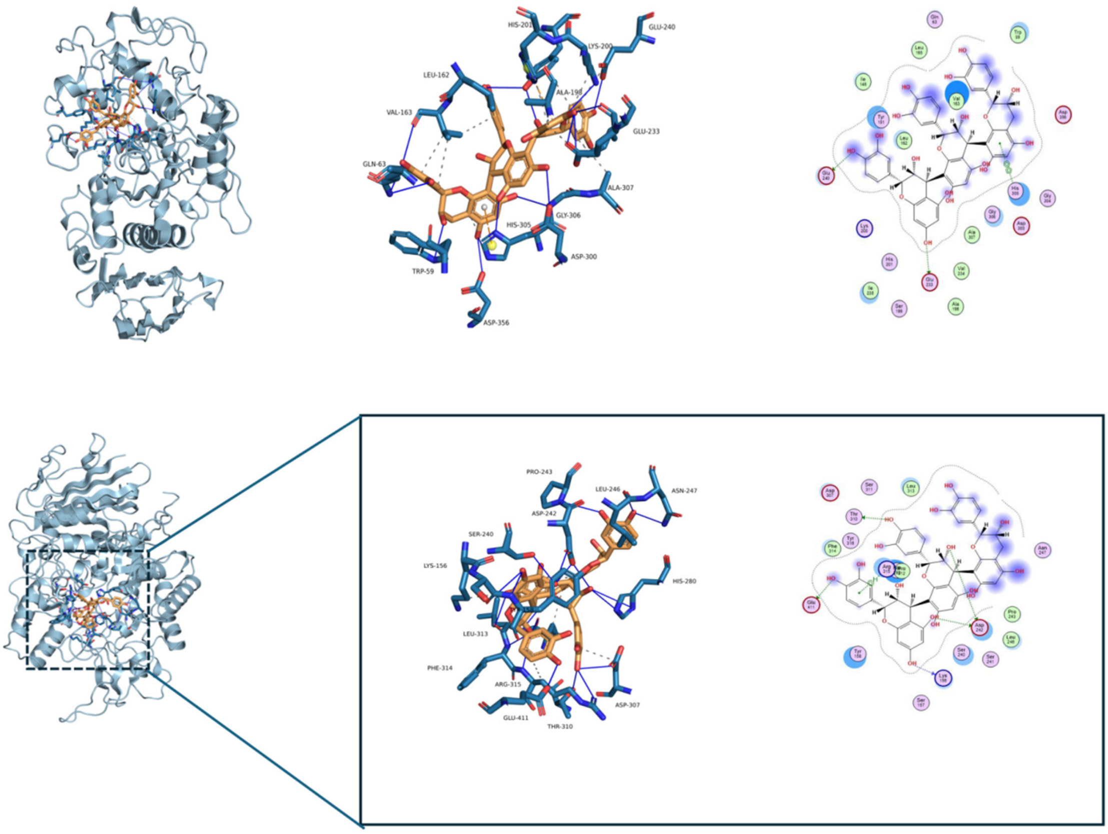

Chlorogenic acid

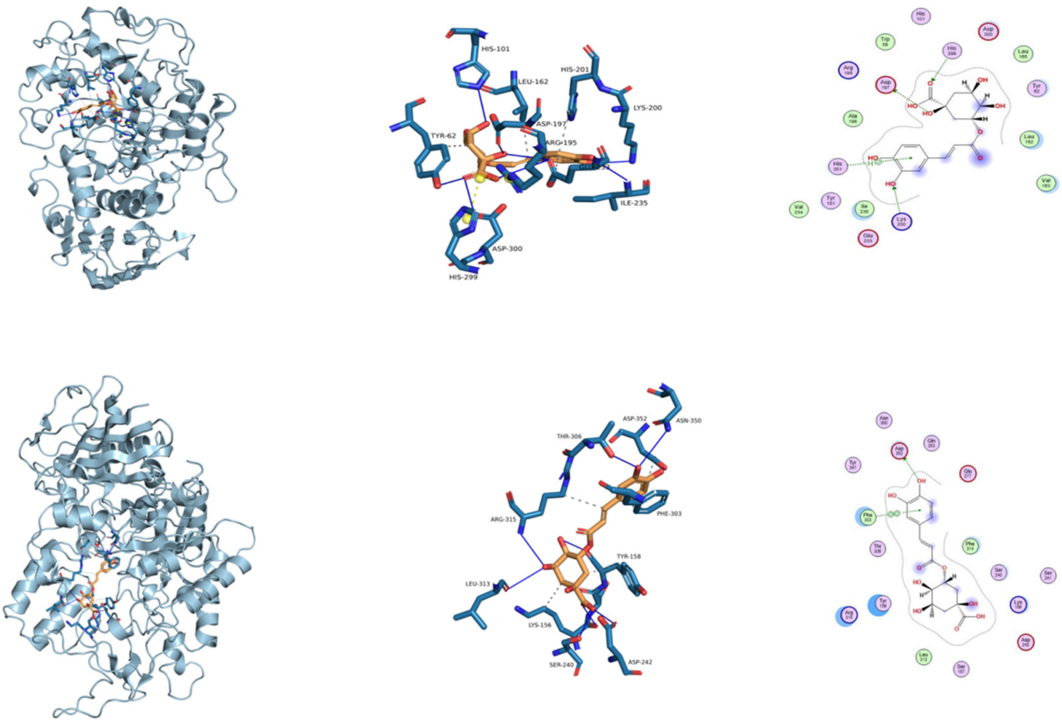

Caffeic acid

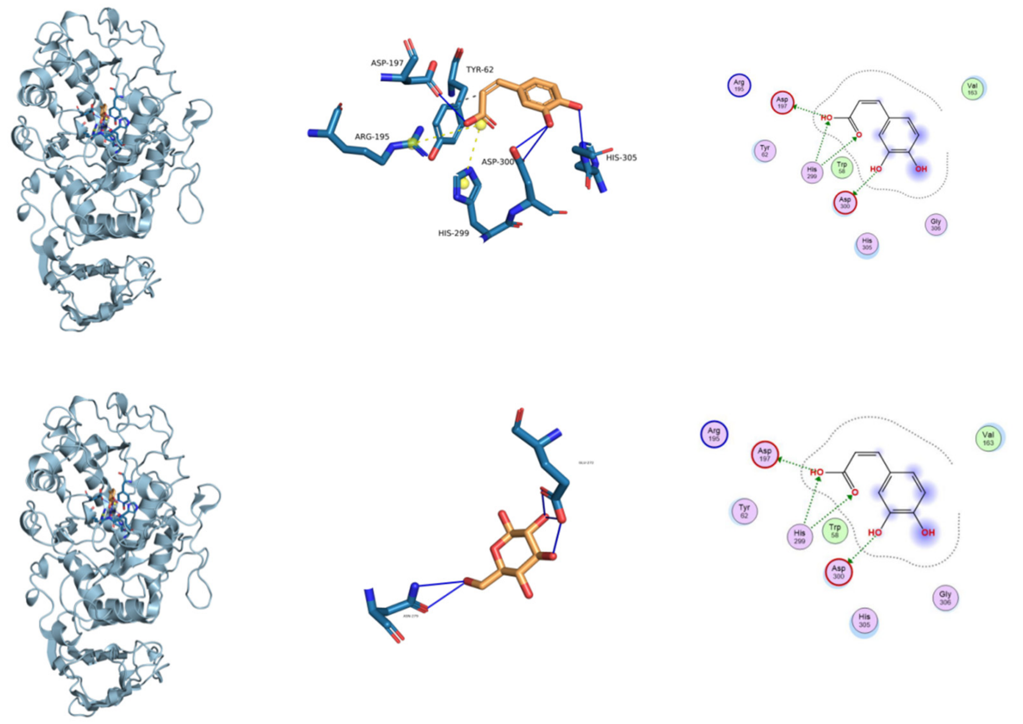

Ellagic acid

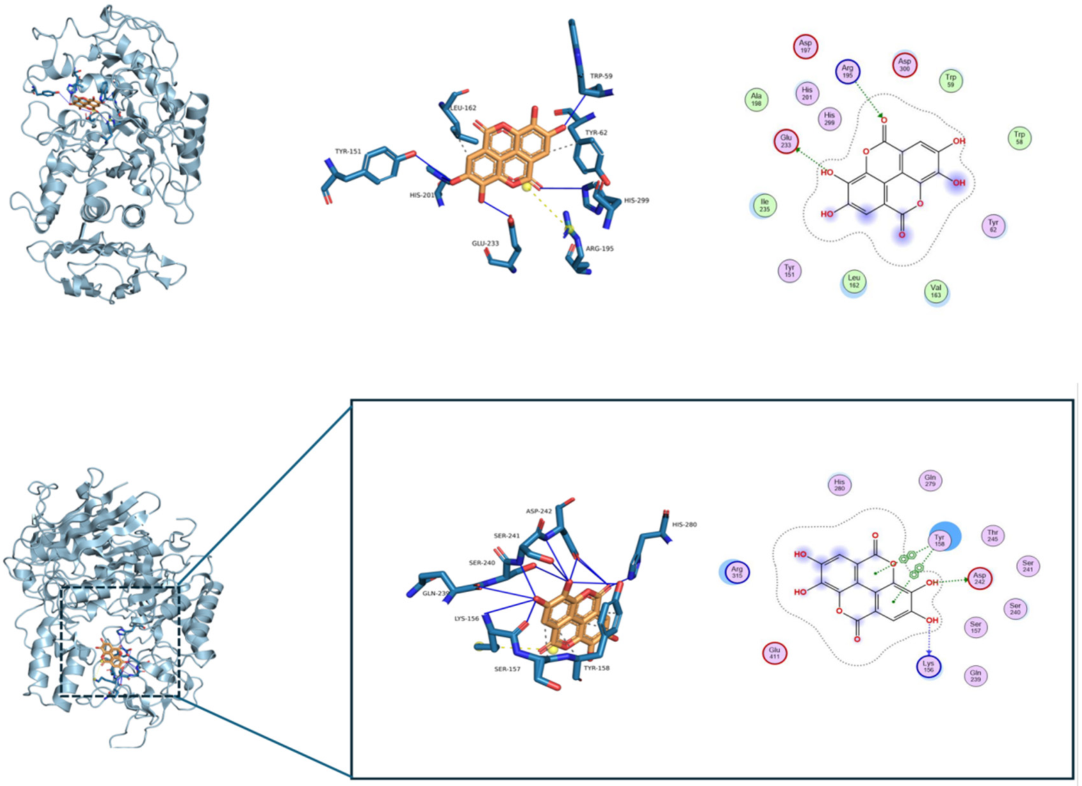

Figure S2. Molecular docking results of eighteen compounds with α-amylase (top) and α-glucosidase (bottom)
